# Dual-Action Therapeutics: DNA Alkylation and Antimicrobial Peptides for Cancer Therapy

**DOI:** 10.3390/cancers16183123

**Published:** 2024-09-10

**Authors:** Celia María Curieses Andrés, José Manuel Pérez de la Lastra, Elena Bustamante Munguira, Celia Andrés Juan, Eduardo Pérez-Lebeña

**Affiliations:** 1 Hospital Clínico Universitario de Valladolid, Avenida de Ramón y Cajal, 3, 47003 Valladolid, Spain; cmcuriesesa@saludcastillayleon.es (C.M.C.A.); ebustamante@saludcastillayleon.es (E.B.M.); 2 Institute of Natural Products and Agrobiology, CSIC-Spanish Research Council, Avda. Astrofísico Fco. Sánchez, 3, 38206 La Laguna, Spain; 3 Cinquima Institute and Department of Organic Chemistry, Faculty of Sciences, Valladolid University, Paseo de Belén, 7, 47011 Valladolid, Spain; celia.andres.juan@uva.es; 4 Sistemas de Biotecnología y Recursos Naturales, 47625 Valladolid, Spain; info@glize.eu

**Keywords:** chemotherapy, alkylating agents, DNA alkylation, cancer, DNA damage and mutations

## Abstract

**Simple Summary:**

Conventional cancer treatments, based on chemotherapy and radiotherapy, are often effective but suffer from serious side effects and a potential risk of resistance. Dual therapies, combining DNA alkylating agents and antimicrobial peptides, are generating great interest. Within chemotherapies, a frequently used mechanism is DNA alkylation, inducing DNA damage and subsequent cell death. Antimicrobial peptides, in turn, have demonstrated their efficacy as anticancer agents due to their ability to selectively alter cancer cell membranes. In this review, our aim has been to explore the synergistic potential of these two therapeutic modalities when used together.

**Abstract:**

Cancer remains one of the most difficult diseases to treat, requiring continuous research into innovative therapeutic strategies. Conventional treatments such as chemotherapy and radiotherapy are effective to a certain extent but often have significant side effects and carry the risk of resistance. In recent years, the concept of dual-acting therapeutics has attracted considerable attention, particularly the combination of DNA alkylating agents and antimicrobial peptides. DNA alkylation, a well-known mechanism in cancer therapy, involves the attachment of alkyl groups to DNA, leading to DNA damage and subsequent cell death. Antimicrobial peptides, on the other hand, have been shown to be effective anticancer agents due to their ability to selectively disrupt cancer cell membranes and modulate immune responses. This review aims to explore the synergistic potential of these two therapeutic modalities. It examines their mechanisms of action, current research findings, and the promise they offer to improve the efficacy and specificity of cancer treatments. By combining the cytotoxic power of DNA alkylation with the unique properties of antimicrobial peptides, dual-action therapeutics may offer a new and more effective approach to fighting cancer.

## 1. Introduction

Chemotherapy uses drugs to kill cancer cells. Chemical compounds are used to selectively shrink or destroy a tumor or at least limit its growth. Compared to surgery and radiotherapy, chemotherapy has the advantage that the drugs are distributed in most tissues of the body after oral or intravenous administration and can, therefore, also destroy tumor cells that metastasize or are located in protected areas such as the brain [1].

Today, many types of chemotherapy or chemotherapeutic drugs are used to fight cancer, which can be administered as combination treatments or individually or together with other treatments. These drugs vary greatly in their chemical composition, how they are prescribed and administered, how effective they are in treating certain types of cancer, and what side effects they may have. Cancer encompasses a large group of diseases characterized by the development of abnormal cells that divide and grow uncontrollably in any part of the body [2].

The chemical compounds used in chemotherapy are cytotoxic, meaning they are designed to kill cancer cells, which have the property of multiplying faster than normal cells, but they also act on normal tissue, meaning they are able to stop the replication or growth of cancer cells and cause their death. A specific type of drug is used, with the number of treatments and the length of treatment depending on the type of cancer and the patient’s particular circumstances [3].

Cancer drugs do not all work in the same way, and it is also important to consider the side effects they can cause depending on the dose being administered. This leads to side effects that vary from patient to patient. The most common are bone marrow suppression, hair loss, fatigue, nausea, and vomiting. These side effects reflect the fact that the cells of the bone marrow, gastrointestinal tract, and hair follicles divide faster than most normal tissues [4].

Cancer treatment usually involves more than one approach, and the strategy chosen depends on the type of cancer, the presence of a biomarker, and the stage of tumor progression. Some cancer patients require only one type of treatment, while others may need several, administered at different times and in specific combinations [5].

A comparison between targeted therapy and chemotherapy shows that both use drugs to attack and destroy cancer cells. However, targeted therapy is designed to act specifically on proteins with mutations that are unique to cancer cells, reducing the potential damage to normal tissue. Targeted therapy is often used in combination with other treatments. Currently, it is not an ideal method for eliminating cancer, as it still requires the use of strong chemicals that can also cause side effects such as hair and skin problems or high blood pressure [6].

## 2. Types of Chemotherapy Drugs

The anticancer drugs currently in use and under development act via different mechanisms to interfere with a number of processes, such as tumor cell growth, motility, survival, or angiogenesis. Chemotherapeutic drugs can be categorized into groups according to their mode of action, chemical structure, and interactions with other drugs. Some drugs act in more than one way and may belong to more than one group.

DNA-damaging agents, such as alkylating agents, platinum derivatives, anthracyclines, and camptothecins, are some of the most commonly used drugs in chemoattractants. Knowledge of ligand–DNA interactions is a key step in the development of new drugs. It is necessary to determine their DNA sequence specificity, the mode of binding to DNA, and the kinetic, dynamic, and structural parameters of binding [7].

The drugs used to fight cancer are called antineoplastic or cytotoxic. They have different mechanisms of action based on agents that are able to mimic properties of biomolecules to disrupt growth or destroy cells and are characterized by being non-selective, i.e., they act on both cancer cells and healthy cells [2].

**Figure 1:**
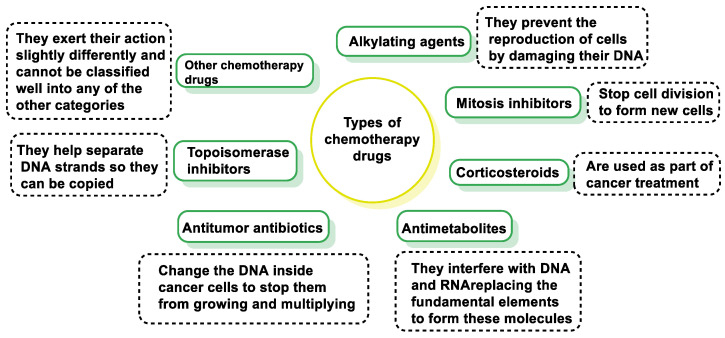
Classification of oncological drugs and mechanism of action of each drug type.

To classify anticancer drugs, the following characteristics can be considered: (i) chemical structure, (ii) function, and (iii) interaction with other drugs [8]. The treatments used are based on the action of the pharmacological groups shown in Figure 1.

Different antineoplastic drugs can act on one or more phases of the cell cycle or on the mechanisms controlling cell proliferation. The same drug may have more than one mode of action on the tumor cell, although usually one of them predominates. The classification currently used is based on the target or site of action of the antineoplastic [9,10] and is shown in Table 1.

Within the antineoplastic drugs, alkylating agents are commonly used. These treatments are able to intervene in one or more phases of the cell cycle or in the mechanisms that control cell proliferation, and at least two drugs are used to act on different therapeutic targets and avoid the emergence of resistance.

Unfortunately, due to the lack of specificity of cytotoxic treatments, there are a variety of adverse effects, the most common of which are rapid death of healthy cells, bone marrow depression, anemia, nausea, vomiting, alopecia, impaired wound healing, growth failure in children, necrosis, sterility, and possible long-term carcinogenesis [11].

Anthracycline derivatives such as doxorubicin (DOX) are another of the most widely used compounds and are the main component of cancer treatment regimens currently used in clinical practice. However, the precise mechanisms of their action are not fully understood [12].

The anticancer activity of DOX may be attributed to the drug’s ability to intercalate into DNA, inhibit topoisomerase II, alter mitochondrial function, and enhance free radical generation and oxidative damage. The precise mechanisms of action of DOX are complex and still relatively unknown [13,14,15].

Like other anthracyclines, DOX intercalates DNA through the formation of hydrogen bonds with guanines in adjacent GC base pairs [16,17].

DOX induces DNA damage through three main mechanisms: DNA adduct formation, single-strand break (SSB) induction, and double-strand break (DSB) induction, in which DNA strands remain attached to trapped topoisomerase enzymes through DNA–protein cross-linking (DPC) and DOX intercalation into the DNA molecule. One possible mechanism postulated to explain the effect of DOX in malignant cells is that DOX intercalation into DNA unwinds the molecule and results in positive supercoiling of the DNA helix [18,19]. There is also evidence that DOX increases the turnover of nucleosomes surrounding active gene promoters due to its intercalation and induces changes in DNA topology [20]. It is possible that the DNA unwinding that occurs as a result of DOX intercalation provides a significant amount of positive torsional stress that destabilizes nucleosomes [21].

Topoisomerase II inhibitors are among the most effective antitumor drugs used in the treatment of human tumors [22].

Lemke K. et al., 2005, have described the antitumor drug 5-dimethylaminopropylamino-8-hydroxytriazoloacridinone, C-1305, which belongs to the group of acridine derivatives, binds to DNA by intercalation, and is an inhibitor of DNA topoisomerase II [23]. C-1305 induces structural perturbations in regions of DNA with three adjacent guanine residues. C-1305 binds to DNA by intercalation and induces unusual structural changes in regions of DNA with guanine triplets.

A study of direct C-1305-mediated microtubule stabilization as the central mediator leading to apoptosis was performed in 2020. Furthermore, C-1305 was found to promote G2 cell cycle arrest by modulating gene expression, indicating that C-1305 is the first microtubule-stabilizing agent identified together with topoisomerase II inhibitory activity [24].

## 3. Binding of a Ligand to DNA

**Figure 2:**
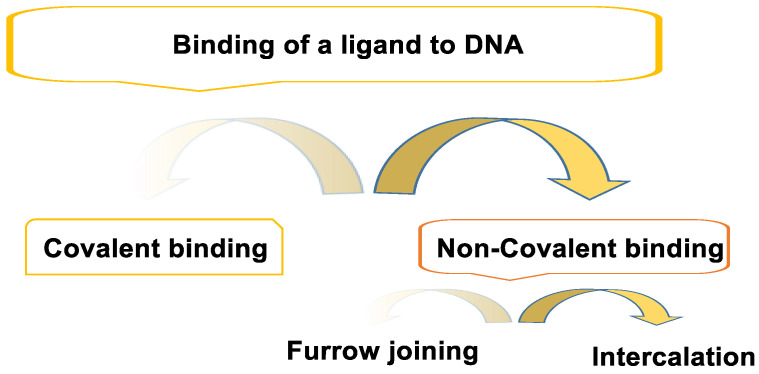
Modes of ligand binding to DNA. DNA can bind to small molecules or drugs through covalent or non-covalent interactions. Covalent binding in DNA can be irreversible and can lead to inhibition of all DNA processes that subsequently lead to cell death. Covalent interactions lead to permanent changes in the structure of nucleic acids. Non-covalent interaction of molecules with DNA can be due to electrostatic interaction, intercalation, and groove binding.

The binding of a ligand to DNA can be classified into covalent and non-covalent binding (Figure 2).

### 3.1. Non-Covalent Bond

Non-covalent bonds are hydrogen bonds, electrostatic interactions, or non-covalent bonds with ethidium bromide and quinacrine. Non-covalent binding can occur in different ways, e.g., groove binding and intercalation. Agents that interact with non-covalent DNA (groove, intercalators, and external binders) are generally considered less cytotoxic than agents that produce covalent DNA adducts and other types of DNA damage. The non-covalent mode of binding is reversible and is generally preferred over covalent adduct formation in terms of drug metabolism and toxic side effects. In addition, drugs that interact with non-covalent DNA can alter DNA conformation, torsionally stress DNA, disrupt protein–DNA interactions, and potentially lead to DNA strand breaks [25].

### 3.2. Covalent Bonding

The first cancer chemotherapeutics were those that formed a covalent bond by alkylating the nitrogenous bases of DNA. This binding is irreversible and leads to a complete inhibition of DNA processes and subsequent cell death [26].

The alkylation of adenine and guanine at the nitrogen atom and of guanine at the oxygen atom can easily be caused by alkylating chemicals. If these errors are not corrected by DNA repair processes, cell mutations can occur. In medicine, DNA alkylation is used beneficially in the treatment of cancer. Alkylating chemicals impair DNA replication and can, therefore, cause cell death. This effect is particularly pronounced in rapidly dividing cells, such as cancer cells [27].

## 4. DNA Alkylating Agents

Alkylating agents prevent the proliferation of cells by damaging their DNA. These drugs work at all stages of the cell cycle and are used to treat many different types of cancer, including lung, breast, and ovarian cancer, as well as leukemia, lymphoma, Hodgkin’s disease, multiple myeloma, and sarcoma.

As these drugs damage DNA, they can affect the bone marrow cells that form new blood cells. The risk of leukemia from alkylating agents is dose-dependent, i.e., the risk is lower at lower doses but increases as the total amount of the drug is increased. The risk of leukemia after administration of alkylating agents is highest about 5 to 10 years after treatment.

The characteristic toxicities of alkylating agents are (i) hematopoietic toxicity, (ii) gastrointestinal toxicity, and (iii) gonadal and central nervous system (CNS) toxicity. However, each of these agents has a characteristic set of toxicities that are determined by the reactivity, metabolism, and distribution of the drug.

**Figure 3:**
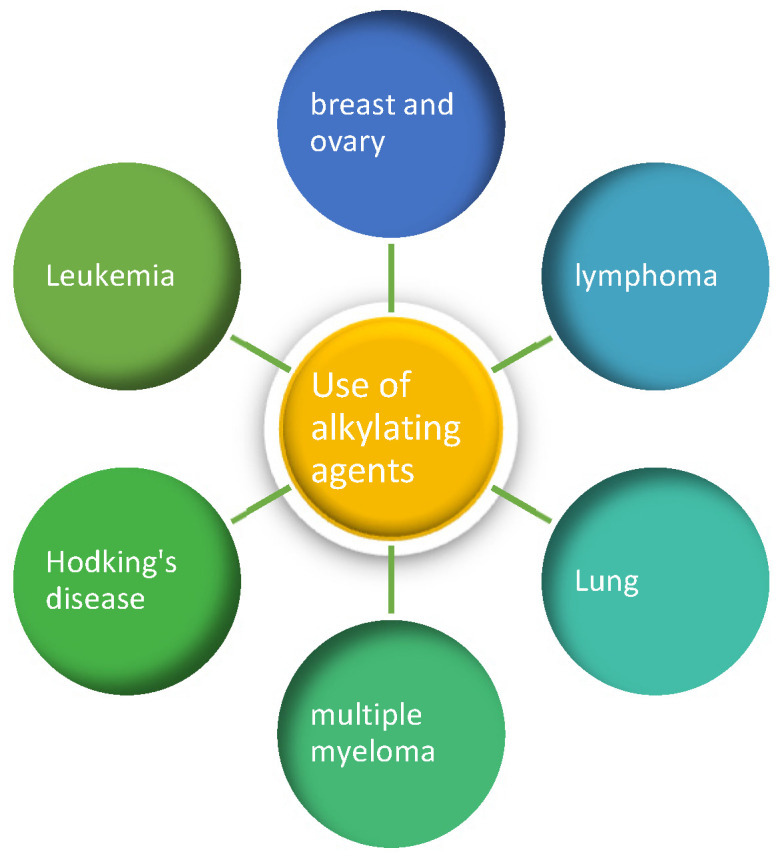
Types of cancers treated with alkylating agents.

Despite their toxicity, DNA alkylating drugs remain the cornerstone of cancer therapy. DNA-targeting alkylating agents were among the first anticancer drugs and are still the most commonly used in chemotherapy today. Alkylating agents used as anticancer drugs are able to react with DNA and proteins and disrupt cell function at several stages of the cell cycle, either killing the cell or preventing it from growing. The National Cancer Institute defines an alkylating agent as a type of drug that is used to treat cancer by interfering with the cell’s deoxyribonucleic acid (DNA) and inhibiting cancer growth. Although alkylating agents can be used to treat most types of cancer, they are usually most useful in treating slow-growing cancers [28]. Alkylating agents are used to treat the types of cancer shown in Figure 3.

**Figure 4:**
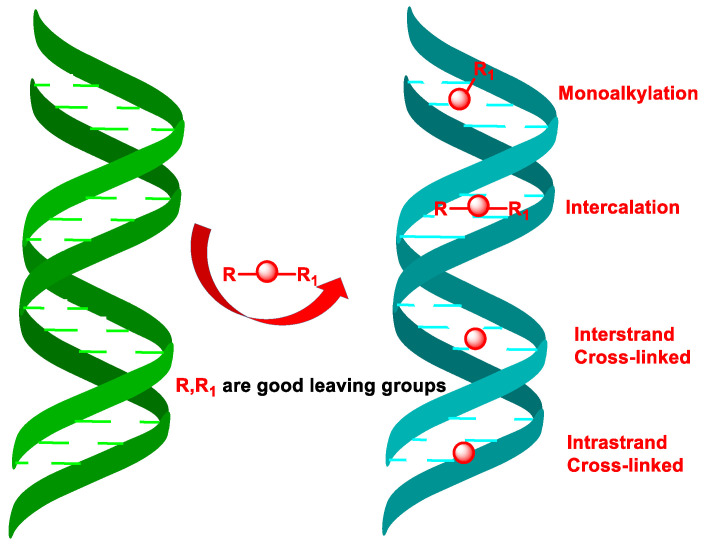
Different types of links produced on DNA by bis-alkylating agents.

Based on the number of alkyl groups in their molecule, alkylating agents are classified as monofunctional, bifunctional, and polyfunctional; the last two are the most effective antitumor agents. Alkylating compounds cause mono-alkylation or can intercalate into DNA. In addition, bis-alkylating compounds can form bridges within a single DNA strand or between two complementary DNA strands (Figure 4), as well as cross-links between DNA and associated proteins [29].

The structure and dynamics of DNA are strongly affected by the alkylation of its bases, leading to different types of effects: (i) prevention of DNA replication, (II) DNA fragmentation, (iii) induces nucleotide mismatches by disrupting the normal hydrogen bonds between bases, (iv) bis-alkylation causes intra-catenary cross-linking, (v) cross-linking between two complementary DNA strands preventing their separation during DNA replication or transcription, (vi) cross-linking between DNA and associated proteins. Bifunctional alkylating compounds are more cytotoxic than monofunctional ones. Many anticancer drugs interact with DNA by intercalation, whereby compounds containing aromatic or heteroaromatic rings are inserted between neighboring base pairs perpendicular to the helix axis without altering the overall stacking pattern due to Watson–Crick hydrogen bonding [30,31].

**Figure 5:**
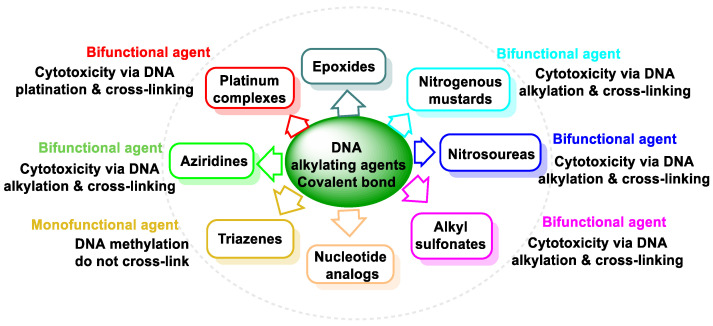
Classification of antineoplastics–alkylating agents according to the reactive groups. They may act as monofunctional agents or may form bifunctional derivatives by forming cross-links (inter- or intra-chain) in DNA or between DNA and proteins.

Based on the chemical structure of the reactive groups in antineoplastic drugs, they are categorized into the following groups: nitrogen mustards, nitrosoureas, alkyl sulphonates, triazines, and ethylenimines, and this group of covalent bonds also includes platinum compounds (Figure 5). Alkylating chemotherapies have been used for years and can be effective against many types of cancer. However, because they attack all cells, the side effects can be severe. The goal in using these older alkylating neoplastic agents is to carefully titrate the dose to ensure that the cancer cells are attacked the most while minimizing damage to non-cancerous cells.

An alkylating agent is defined as a compound capable of covalently bonding an alkyl group to a biomolecule under physiological conditions (aqueous solution, 37 °C, pH 7.4); DNA alkylating agents interact with resting and proliferating cells at any stage of the cell cycle but are most cytotoxic in late G1 and S phase, as there is insufficient time to repair DNA damage. Alkylating agents, especially nitrogen-based alkylating agents, are commonly used in the treatment of hematologic and solid malignancies. They exert their antineoplastic effect in all phases of the cell cycle and prevent tumor cells from proliferating.

**Figure 6:**
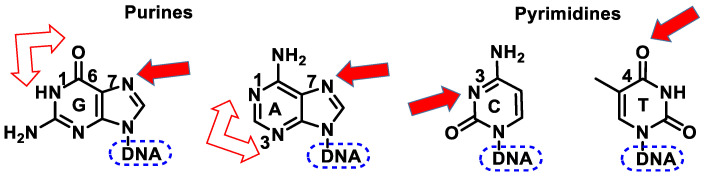
Arrows indicate DNA base alkylation sites.

All nitrogen and oxygen atoms of these bases are nucleophilic, with the exception of the nitrogen atoms involved in nucleoside binding (N9 or N1 in purines or pyrimidines), therapeutically useful drugs always behaving as carbon electrophiles. Alkylating agents are electrophilic and bind covalently to electron-rich functional groups, such as the N-7, N-1, and O-6 positions of guanine [32]. Other nucleobases that can be alkylated and the atomic positions where alkylation preferentially occurs are the N-1, N-3, and N-7 positions of adenine, the N-3 position of cytosine, and the O-4 position of thymidine (Figure 6).

Nitrogen-based alkylating agents, which are frequently used to treat hematological and solid malignancies, often in combination with other chemotherapeutic agents, exert their antineoplastic effect at all stages of the cell cycle and prevent the replication of tumor cells. During this alkylation process, an alkyl residue is introduced into the DNA bases, a change that leads to fragmentation of the DNA by DNA repair enzymes that attempt to replace the alkylated base [33].

## 5. Nitrogen Mustards

One of the largest and most diverse groups of drugs are those that affect the structure and function of DNA. Part of the reason that DNA-interfering drugs are the largest group of agents in clinical use today is that nitrogen mustard was the first type of chemotherapeutic agent to be discovered. They have been used for more than 60 years [34]. Nitrogen mustard acts on the kidneys, heart, bladder, central nervous system, and gonads.

**Figure 7:**
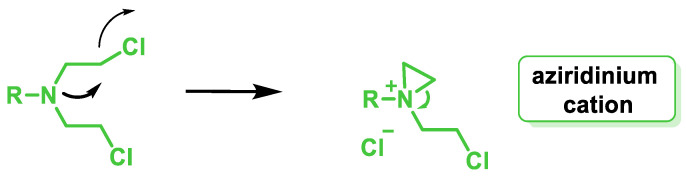
In situ generation of an aziridinium cation.

After ingestion, the alkylating agent is metabolized to a highly reactive aziridinium derivative that alkylates DNA and inhibits DNA reduplication. There are several generations of nitrogen-based alkylating drugs. The mustard compounds generated from the nitro group contain the bis(2-chloroethyl) group, which is the major chemical constituent, and all have a common mechanism of action, namely the in situ generation of a highly electrophilic aziridinium cation (Figure 7) by an intramolecular nucleophilic substitution reaction [35].

### 5.1. Aliphatic Mustard

**Figure 8:**
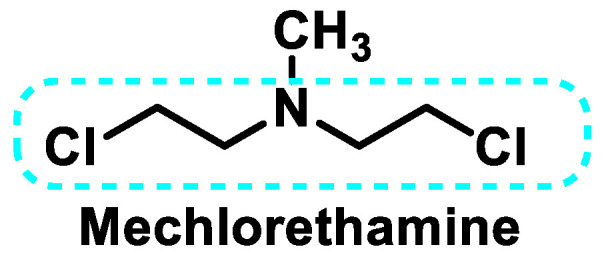
Mechlorethamine structure.

Mechlorethamine (Figure 8) was the first antitumor agent developed during the First World War as part of the mustard gas study. Due to its short half-life, it must be prepared and administered immediately. It can only be administered intravenously. At physiological pH, mechlorethamine is converted via the free base into a relatively stable aziridinium ion, which in turn reacts with available nucleophilic centers. Mechlorethamine has been replaced by new derivatives to improve selectivity and specificity in the fight against cancer cells [36].

### 5.2. Phosphamide Nitrogen Mustard

**Figure 9:**
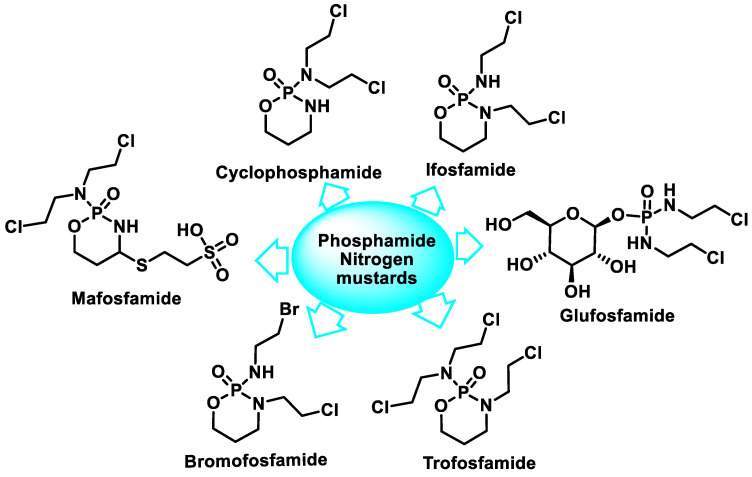
Phosphamide nitrogen mustard.

The oxazaphosphorins have complex pharmacokinetics, and metabolism is crucial for their cytotoxic activity. There are several classes, such as cyclophosphamide, iphosphamide, maphosphamide, trophosphamide, bromophosphamide, and gluphosphamide (Figure 9).

**Figure 10:**
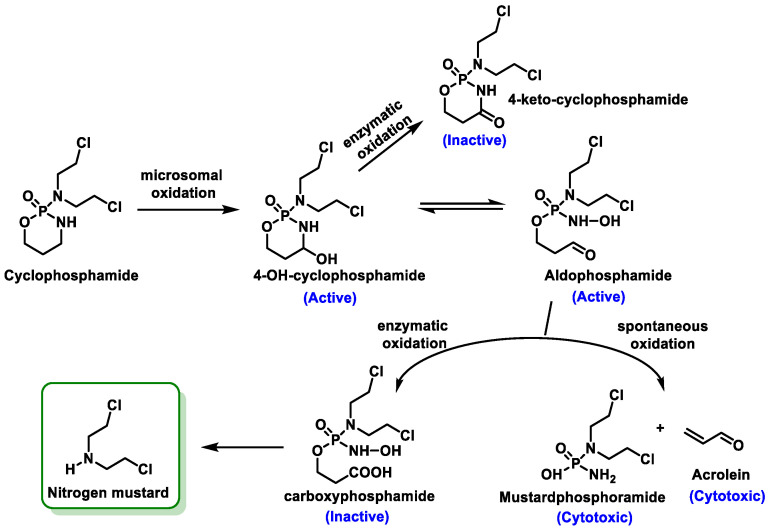
Biotransformation mechanism of cyclophosphamide.

Cyclophosphamide is the most commonly used alkylating agent. It is a prodrug that is converted to its active form after metabolism by cytochrome P-450. Hydroxylation of the carbon at position 4 is the first metabolic reaction of cyclophosphamide and iphosphamide. This reaction occurs in the liver microsomes via the enzyme systems of the 2A6, 2B6, 2C, 3A4, and 3A5 subfamilies of the cytochrome P450 (CYP) enzyme complex. The main metabolite of this reaction is 4-hydroxycyclophosphamide or 4-hydroxyphosphamide, which is in tautomeric equilibrium with its keto form, aldophosphamide. From aldophosphamide, a β-elimination reaction produces phosphoramide mustard with an alkylating effect and acrolein, a urotoxic compound responsible for hemorrhagic cystitis [37] (Figure 10).

Its main toxic effects are myelosuppression for 10–14 days, hair loss, nausea, and vomiting. It can also cause hemorrhagic cystitis due to the effect of some of its metabolites, such as acrolein, on the bladder epithelium; high fluid intake for 24 to 48 h and Sodium-2-mercapto-sulphonate (mesna) to chelate acrolein and avoid this complication. It is a drug widely used in oncology and is part of polychemotherapy regimens, as its efficacy has been demonstrated in various neoplasms (leukemias, lymphomas, breast cancer, ovarian cancer, and sarcomas). It is also part of the main induction regimens prior to bone marrow transplantation.

Isophosphamide is a drug similar to cyclophosphamide but requires higher doses to achieve the same antitumor effect. It is only administered intravenously, always with sufficient fluid intake and mesna as a prophylactic measure [38]. Iphosphamide is a prodrug that must be converted into a cytotoxic metabolite (4-hydroxy derivatives) by the action of CYP3A4 isoforms [39]. Iphosphamide generates seven-atom DNA cross-links (compared to five-atom cross-links for cyclophosphamide).

**Figure 11:**
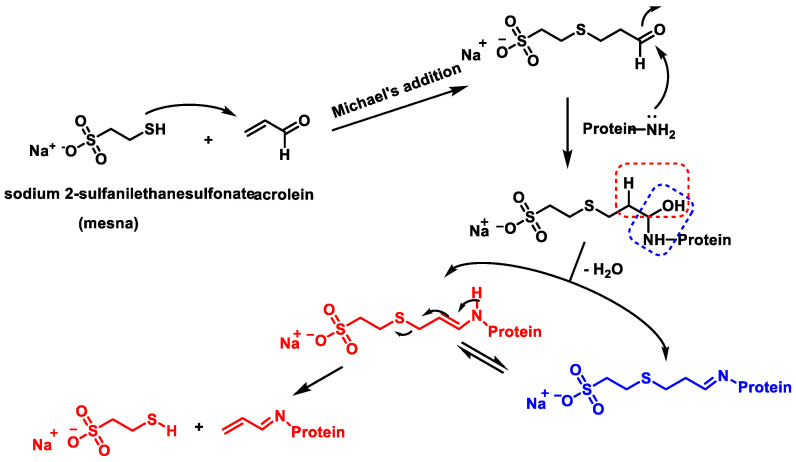
Mechanism of acrolein toxicity.

Acrolein, a metabolite of cyclophosphamide and iphosphamide, is a highly toxic aldehyde. As a bifunctional electrophile, acrolein reacts with protein nucleophiles and DNA via Michael addition and Schiff base formation [40] (Figure 11).

**Figure 12:**
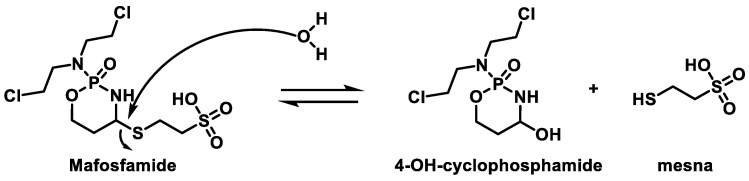
Spontaneous decomposition of Maphosphamide into 4-HO-CP and mesna.

Maphosphamide is a cyclophosphamide analog, an alkylating agent, and an immunomodulator that induces a pro-inflammatory response and increased endothelial cell permeability in vitro [41]. Maphosphamide breaks down spontaneously into 4-OH-CP and mesna (Figure 12). The released 4-OH-CP is in equilibrium with its tautomer aldophosphamide, which can be degraded by β-elimination to generate cytotoxic phosphoramide mustard and acrolein. Thus, maphosphamide is directly active in cells and intracellular fluids. Although the drug is similar to CP, it does not need to be activated by the hepatic enzyme system [42].

Maphosphamide has significant in vitro activity against leukemia and solid tumor cell lines such as U-937, ML-1, MOLT-4, rhabdomyosarcoma, and MCF-7, and also in vivo against several transplantable mouse tumors, including P388 and L1210 leukemia, B16 melanoma, Lewis lung carcinoma, colon tumor 38, and cyclophosphamide-resistant P388 leukemia.

**Figure 13:**
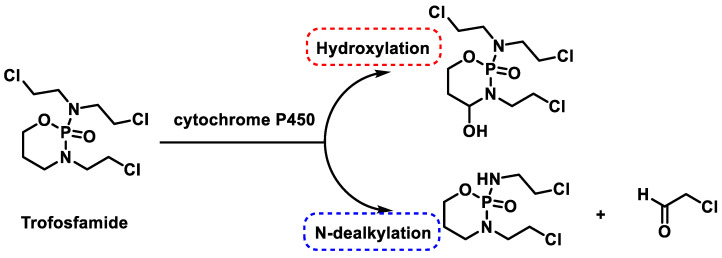
Activation of trophosphamide by cytochrome P450 into oxazaphosphorine mustards. N-dealkylation by cytochrome P450s results in the formation of inactive metabolites and chloracetaldehyde.

Trophosphamide was approved as one of the anticancer drugs in 1973. Like phosphamide and cyclophosphamide, they are non-specific cell cycle alkylating agents that are bioactivated by hepatic cytochrome P450 enzymes through either 4-hydroxylation or N-dechloroalkylation; both are NADPH-dependent [43] (Figure 13).

**Figure 14:**
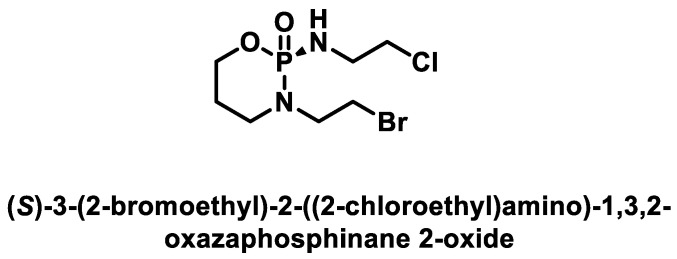
Chemical structure of (S)-(-)-Bromophosphamide Gluphosphamide (β-D-glucoseisophosphoramide-mustard).

Bromophosphamide, an analog of iphosphamide with a bromine substituent, causes less urotoxicity. It acts by cross-linking DNA strands or deoxyribose–protein cross-links and alkali-labile sites in a concentration-dependent manner in HeLa cells [44].

(S)-(-)-bromophosphamide (Figure 14) is an alkylating anticancer drug introduced into clinical practice in the 1980s and used to treat soft tissue sarcomas and a variety of pediatric tumors [45,46].

Gluphosphamide, also known as glucophosphamide (β-D-glucose isophosphoramide mustard), is a glycosidic conjugate between β-D-glucose and the active alkylating moiety of the antineoplastic drug iphosphamide. In gluphosphamide, the active part of iphosphamide is linked to a glucose molecule. Inside the cells, the bond between the glucose and the alkylating agent must be broken in order to release the active substance. Phase III trials are currently underway with this drug, comparing gluphosphamide with 5-FU, both in combination with gemcitabine, for the second-line treatment of metastatic pancreatic cancer [47].

### 5.3. Aromatic Nitrogen Mustards

**Figure 15:**
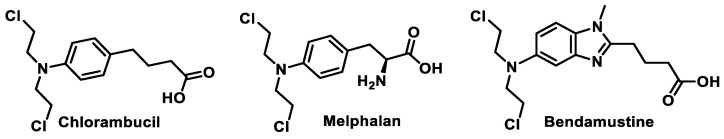
Aromatic nitrogen mustards.

The main aromatic nitrogen mustards are shown in Figure 15.

Melphalan is a phenylalanine derivative of nitrogen mustard and was approved by the FDA in 1964 [48]. It can be administered orally or intravenously. Melphalan is 90% bound to plasma proteins and is actively transported into cells primarily via the L-type amino acid transporter system. The oral bioavailability is 50–80%, and the half-life can be up to 8 h. It is partially excreted in the feces and partially in the urine. Melphalan was developed for the treatment of melanoma because it selectively targets tumor cells that actively use tyrosine. However, it has shown little effect in this neoplasm. Currently, its main indication is multiple myeloma. It has been shown to be effective at conventional doses in ovarian cancer and lymphoma and at high doses in breast cancer and acute myeloid leukemia.

Chlorambucil was approved by the FDA in 1957 [48]. It is stable in aqueous solution so that it is almost completely absorbed after administration and is rapidly and completely excreted from the blood. It is metabolized in the liver and has the alkylating metabolite PAAM (phenylacetic acid mustard). Adverse effects are uncommon, with the exception of bone marrow suppression. However, it can cause a severe generalized rash, which may develop into Stevens–Johnson syndrome or toxic epidermal necrolysis. In the event of a rash, further treatment with chlorambucil is contraindicated. This drug is indicated for the palliative treatment of chronic lymphocytic leukemia, malignant lymphoma, lymphosarcoma, follicular giant lymphoma, and Hodgkin’s disease [49].

Bendamustine: N-methylbenzimidazole replaced the benzene ring of chlorambucil to form bendamustine. The drug was approved by the FDA in 2008, although it was discovered in 1960. The incorporation of the benzimidazole ring gives the molecule the properties of a purine analog [50].

### 5.4. Steroid-Coupled Nitrogen Mustards

Alkylating agents have high toxicity and low specificity for alkylating agents, but when coupled with a steroid, they are more effective in improving the therapeutic profile. Steroid-coupled nitrogen mustards utilize the action of both the alkylating agent and a steroid for selective uptake or a combined anticancer effect. The steroid acts as a biological carrier, allowing the nitrogen mustard component to be easily transported and increasing the uptake of the mustard through the lipid bilayer of cells [51]. The result is a more effective, more selective, and less toxic antineoplastic treatment. Saha et al., 2018, have shown that steroid-bound mustard enhances the damaging effect on a specific DNA sequence and achieves better selectivity and lower toxicity compared to nitrogen mustard itself [51,52]. Some of the most important drugs in this class are listed below:

Estramustine phosphate sodium: Estramustine has a β-estradiol moiety attached to a nitrogen mustard moiety (mechlorethamine) via a carbamate bridge. Chemically, it is estra-1,3,5(10)-triene-3,17β-diol-3-bis(2-chloroethyl)-carbamate. Due to the stability of the carbamate bridge, which is not cleaved to release the nitrogen mustard, the compound has no alkylating activity.

**Figure 16:**
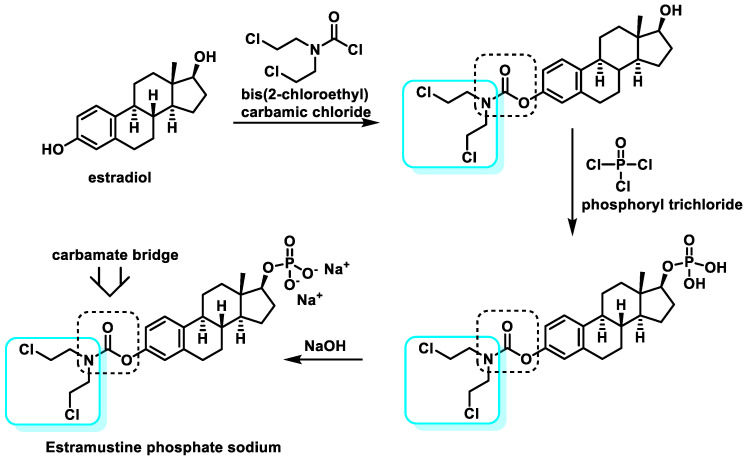
Chemical synthesis of Estramustine phosphate sodium.

In 1986, estramustine sodium phosphate was synthesized by condensation of estradiol with bis(2-chloroethyl) carbamyl chloride, followed by phosphatidylation with phosphorus oxychloride and finally treatment with NaOH in EtOH [53] (Figure 16).

The compound is dephosphorylated during absorption and broken down into estradiol, estrone, and estramustine as the main metabolites in the blood. The estradiol component increases the uptake of the alkylating agent into the target cells. Estramustine phosphate is a prodrug marketed for the treatment of advanced prostate cancer.

**Figure 17:**
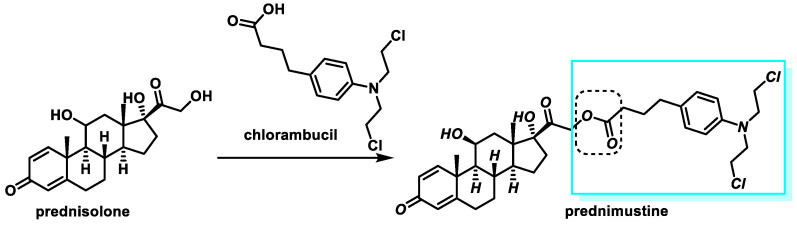
Chemical synthesis of Prednimustine.

Prednimustine: The drug was produced from prednisolone ester (corticosteroid) and chlorambucil (Figure 17). It has both alkylating and corticosteroid properties. Acute leukemia, breast cancer, and lymphocytic leukemia are diseases for which this drug is commonly used [54,55].

**Figure 18:**
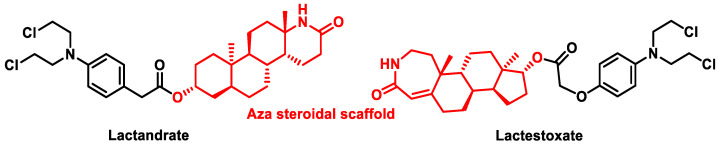
Chemical structure of Lactandrate and Lactestoxate.

Homoazasteroid (lactam steroids): This type of drug is formed by attaching a lactam residue to the esterified form of the alkylating steroid drug. They are effective in various neoplastic tumors and leukemias both in vivo and in vitro. Lactandrate and lactestoxate (Figure 18) showed excellent anticancer results in colon carcinomas. However, the latter showed better results than lactandrate [56]. In 2016, Trafalis et al. reported positive results with four modified forms of lactamsteroids [57].

### 5.5. Peptide-Coupled Nitrogen Mustards

**Figure 19:**
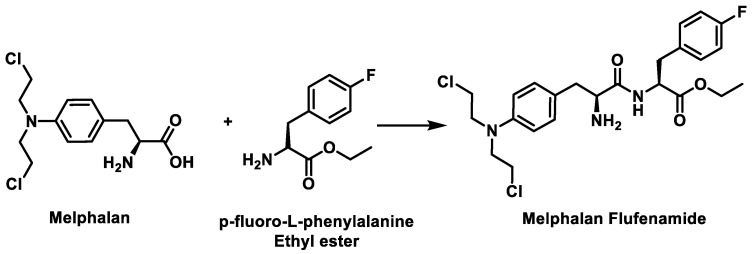
Chemical structure of Melflufen.

Melphalan flufenamide, also known as melphalan flufenamide, is a third-generation drug consisting of the ethyl ester of melphalan and para-fluoro-L-phenylalanine (Figure 19).

**Figure 20:**
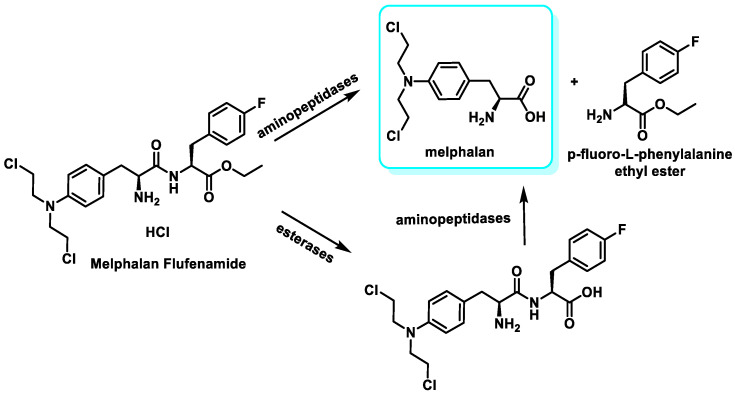
Metabolism of Melflufen hydrochloride.

Being highly lipophilic, it is readily taken up by rare cells (such as myeloma cells) across the membrane barrier, leading to activation by hydrolytic cleavage of the peptide bond using peptidases and esterases overexpressed in multiple myeloma cells to release a toxic cargo into the cells, damaging the DNA and killing the cancer cells [58]. The alkylating component produced during hydrolysis is melphalan, which enters the cell nucleus and causes DNA alteration (Figure 20).

It has a high affinity for solid tumors and multiple myeloma, and phase I/II trials have led to its successful use over time [51]. The drug is effective in third-line treatment in patients who have not previously received alkylating chemotherapeutic agents and in patients who have received melphalan in previous treatments and who are also unsuitable for transplantation [59].

In comparison between melphalan and melflufen, melflufen has a superior cytotoxic effect, higher efficacy at a lower dose, and is effective in treating multiple myeloma cells in which melphalan was not actively involved [60].

Melflufen received accelerated approval in the HORIZON trial on 26 February 2021 [61]. In March 2021, the FDA approved melflufen in combination with dexamethasone for the treatment of adult patients with multiple myeloma. In October 2021, it was withdrawn from the market in the USA after too many deaths occurred in a confirmatory phase 3 study [61].

### 5.6. Mode of Action of Nitrogen Mustards

**Figure 21:**
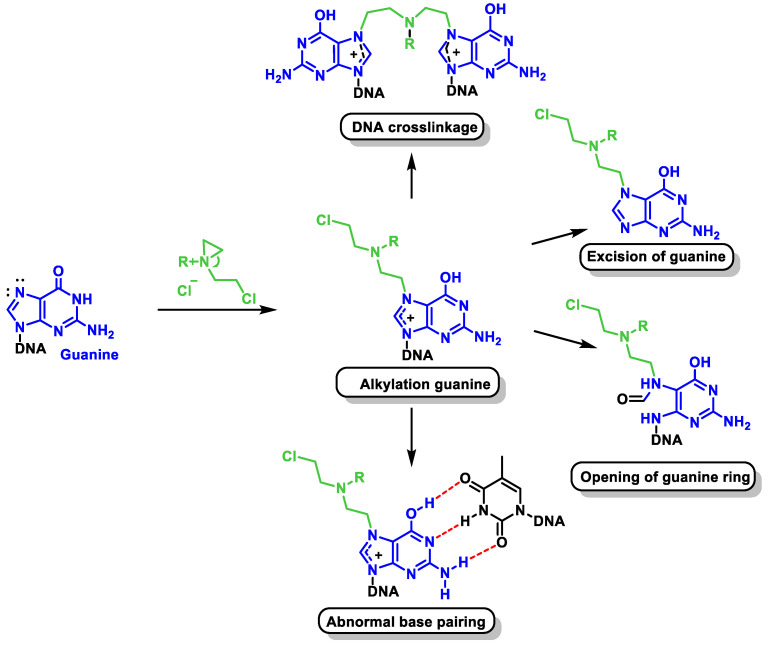
Action of alkylating agents on guanine.

Nitrogen mustard reacts with DNA nucleobases preferentially at the N-7 position of guanine. Their alkylation leads to important changes in the chemical properties of guanine. Since a positive charge is generated on the imidazole ring, the formation of the enolic tautomer is favored, which leads to guanine preferentially pairing with thymine and not with cytosine. In purification processes, hydrolysis leads to local destabilization of the DNA, which would favor strand cleavage. Another consequence is the opening of the imidazole ring, which would also trigger purification (Figure 21). The mechanism of action involves the formation of DNA cross-links, which leads to an inhibition of DNA synthesis and function.

**Figure 22:**
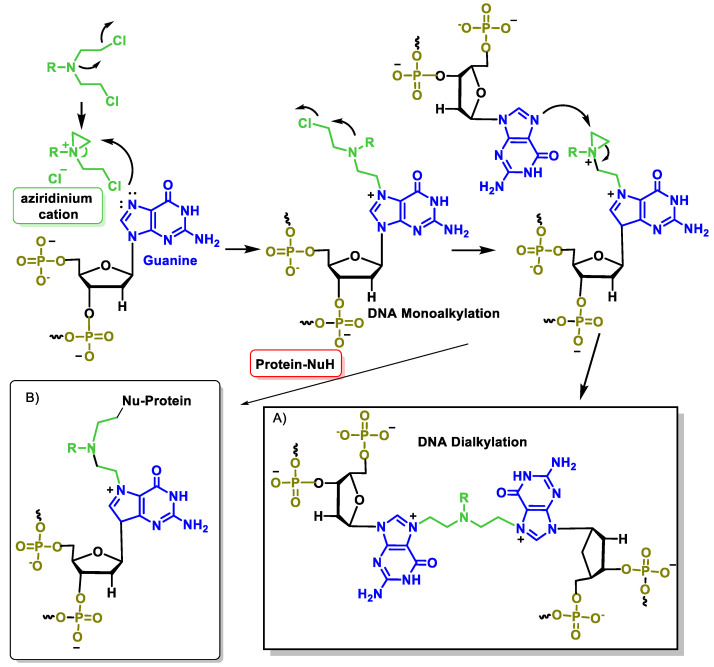
(**A**) DNA monoalkylation and dialkylation mechanism. (**B**) DNA–protein complexes.

The reason for the high reactivity of nitrogen mustard as an alkylating agent under mild conditions is the formation of an aziridinium cation, which acts as an alkylating agent and is formed by intramolecular nucleophilic substitution. The aziridinium cation is highly reactive due to the positive charge of the N and the tension of the three-membered ring. Since nitrogen mustards are bifunctional alkylating agents, one of their cytotoxicity mechanisms is related to the ability of monoalkylated DNA species to form a covalent intercatalytic DNA strand and intracatalytic cross-links or DNA–protein complexes that lead to disruption of replication or transcription (Figure 22).

**Figure 23:**
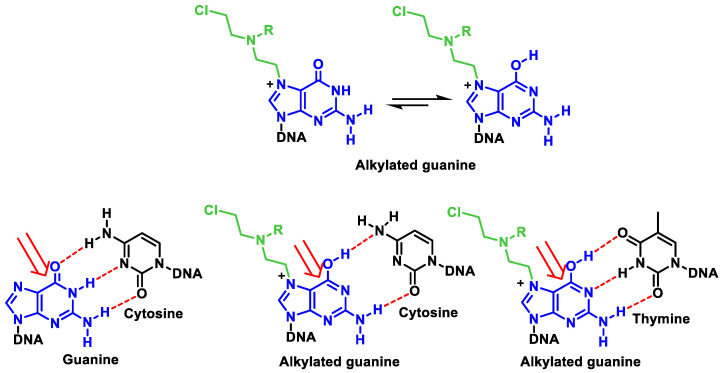
Hydrogen bonding interactions in guanine–cytosine and guanine–thymine pairs.

During alkylation, the normal pairing of the DNA bases between adenine–thymine and guanine–cytosine (Watson–Crick base pairs) is altered. The three hydrogen bonds connecting guanine and cytosine, for example, require the existence of a carbonyl at the C-6 position of the purine derivative. Alkylation at the N-7 position creates a positive charge in this center adjacent to the partial positive charge at C-6, which means that the tautomeric equilibrium shifts to the more stable 6-hydroxy form [62]. This change in the tautomeric form turns the hydrogen bond acceptor groups into hydrogen bond donors and vice versa. This leads to a weakening of the hydrogen bond with cytosine, as only two bonds can be formed, whereas the pairing of the 6-hydroxy species with thymine leads to a more stable complex (three hydrogen bonds). The normal pairing is changed to guanine–non-thymine, which leads to mutations (Figure 23).

**Figure 24:**
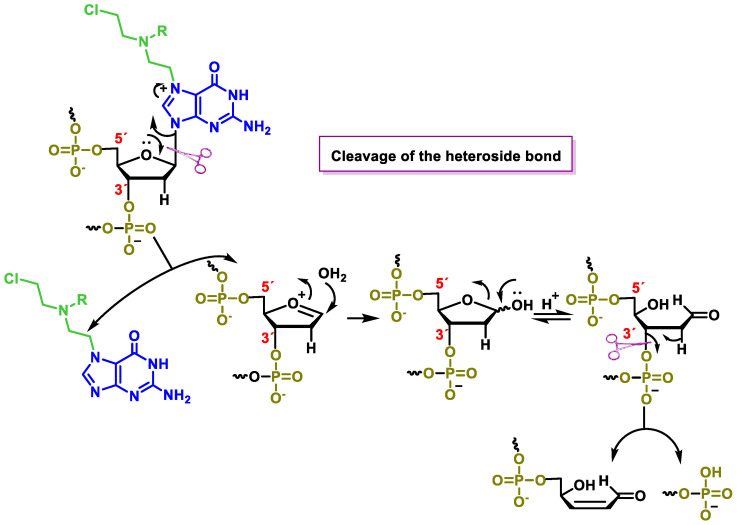
Cleavage of the heteroside bond.

Another consequence of guanine alkylation is an increase in the electrophilicity of the positions adjacent to or conjugated with the positive charge on N-7, which leads to various hydrolytic reactions that alter the DNA structure. This increased electrophilicity allows cleavage of the heteroside bond (Figure 24) and leads to purification of the DNA.

When attacked by water, a cyclic hemiketal forms in equilibrium with the open form, which has a good leaving group (oxygen phosphate) at the β-position with respect to the carbonyl group, facilitating its elimination and leading to DNA fragmentation.

**Figure 25:**
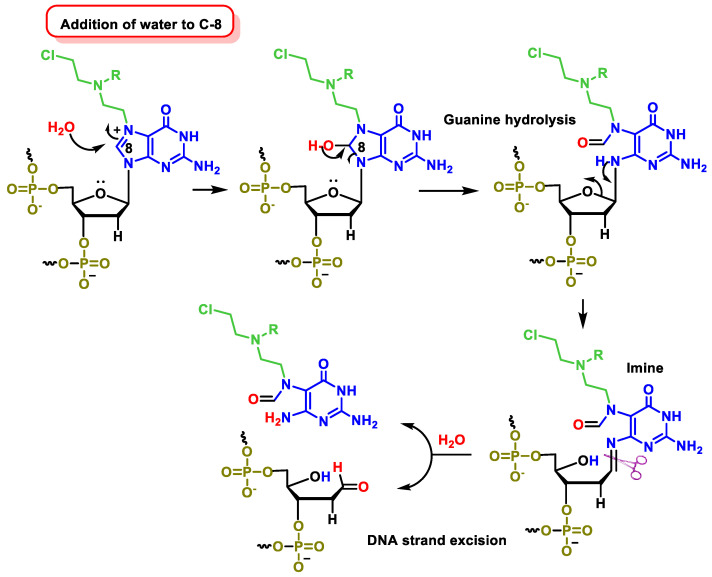
DNA fragmentation that takes place following guanine monoalkylation.

C-8 of the guanine ring becomes more electrophilic as it is close to the positive charge generated in the N-7 alkylation reaction. The addition of water to C-8 provides the hydroxylated intermediate, which opens the purine ring, which then evolves into the imine that develops after hydrolysis to cleave the DNA strand [63] (Figure 25).

## 6. Aziridines

**Figure 26:**
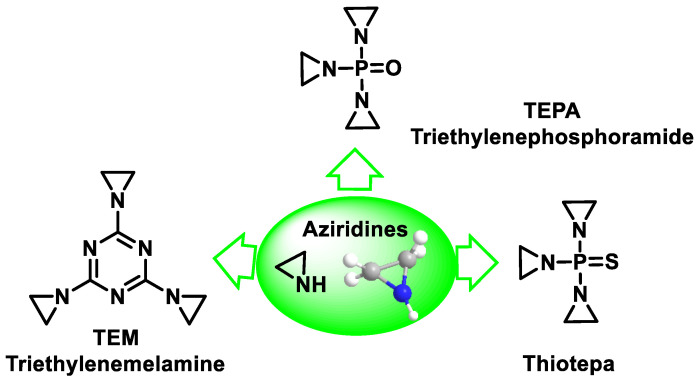
The first compounds derived from aziridines introduced into therapeutics.

Several aziridine derivatives have been described as antitumor agents [64]. It has been found that two aziridine units are required for these compounds to show good activity. It was also found that the presence of a third or fourth aziridine is not required, suggesting that the cytotoxicity is due to a cross-linking mechanism, as observed with nitrogen mustard. Aziridine alkylating agents are characterized by an aziridine ring that is structurally similar to the iminium found in nitrogen mustard. Since they have no charge on the nitrogen, they are less reactive than the nitrogen mustards. It should be noted that the reactivity of the aziridine group increases with protonation and is, therefore, enhanced at low pH. It is assumed that the ring opening of the aziridine ring is responsible for the alkylating activity of the molecule. The mechanism of action of these drugs is less understood. The first compounds of this family to be used are shown in Figure 26.

**Figure 27:**
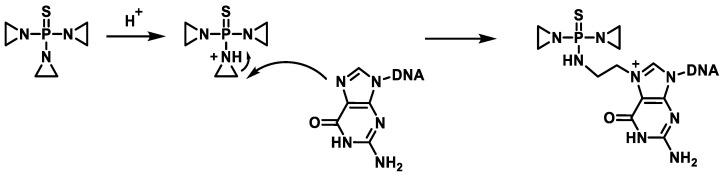
Monoalkylation of guanine by thiotepa.

Thiotepa (tris(1-aziridinyl) phosphine sulfide) is an alkylating agent approved for the treatment of breast cancer, ovarian cancer, and bladder cancer [65,66,67]. Thiotepa is oxidatively desulfurized by hepatic cytochrome P450 to produce TEPA, a less cytotoxic form of the molecule [68,69]. Both compounds have been found in the plasma of patients undergoing this treatment. Thiotepa is currently still used for the treatment of bladder carcinomas. Position 7 of the guanine is the preferred alkylation site of thiotepa (Figure 27).

**Figure 28:**
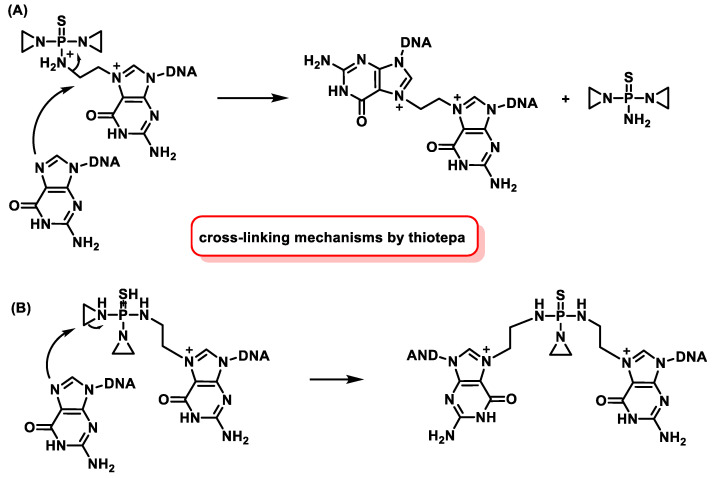
(**A**) alkylation and cross-linking by sequential reaction of a single aziridine group. (**B**) Cross-linking produced by sequential alkylating reactions of two aziridine groups of thiotepa.

In vivo and in vitro studies show that DNA alkylation by ThioTEPA can occur via two pathways, but it is not yet clear which pathway is the exact mechanism of action [70] (Figure 28).

### 6.1. Aziridines Linked to a Benzoquinone System

Alkylating agents containing a quinone moiety require reduction of the quinone structure to activate their alkylating substituents, which are used in the clinic for cancer treatment [71,72].

#### 6.1.1. Natural Aziridinyylbenzoquinone

**Figure 29:**
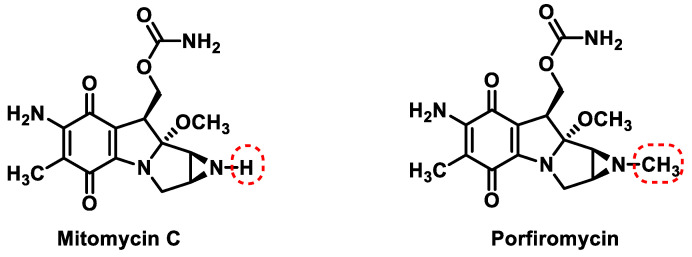
Chemical structure of mitomycins.

Mitomycin C (mutamycin) [73] is a natural antitumor quinone from *Streptomyces caespitosus* [74] that contains a quinone unit and an aziridine (Figure 29). It has been used since the 1960s for the treatment of breast cancer and tumors of the gastrointestinal tract and is considered a cytotoxic agent [75]. Mitomycin C was approved by the FDA in 2020 for adult patients with low-grade urothelial carcinoma of the upper tract.

Porfyromycin is the N-methyl derivative of mitomycin C and is also a natural product. Porfyromycin has been tested in phase III clinical trials for the treatment of head and neck cancer in combination with radiotherapy with acceptable toxicity and stimulating effect [76].

**Figure 30:**
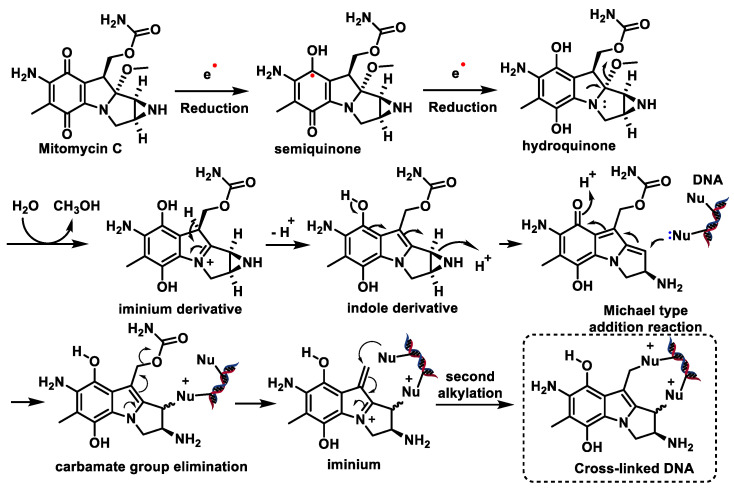
Bioreductive alkylation of DNA by mitomycin C.

Mitomycin C and porfyromycin can be considered as prototypes of reductively activated alkylating agents leading to cytotoxic species [77]. Figure 30 includes two reduction steps with one electron transfer to semiquinone and subsequently to hydroquinone. Both species can initiate the reaction cascade leading to DNA alkylation, but the available evidence points to hydroquinone as the active species [78]. The next step would be the formation of the imine derivative by methanol elimination of the hydroquinone. The next step is the formation of an indole derivative, protonation of the aziridine nitrogen of this derivative, and subsequent elimination with simultaneous opening of the aziridine ring yielding methylquinone methylide. A Michael reaction with nucleophilic DNA groups takes place on this intermediate product, in which the N-2 amino group of the guanine or the N-7 position is involved [79]. With mitomycin, removal of the carbamate group generates an electrophilic iminium, which undergoes a second alkylation by attacking a 2-amino group of guanine, leading to DNA cross-linking [80,81] (Figure 30).

Both inter- and intra-strand cross-linking by mitomycin C have been observed, with the former predominating.

#### 6.1.2. Synthetic Aziridinyylbenzoquinone

**Figure 31:**
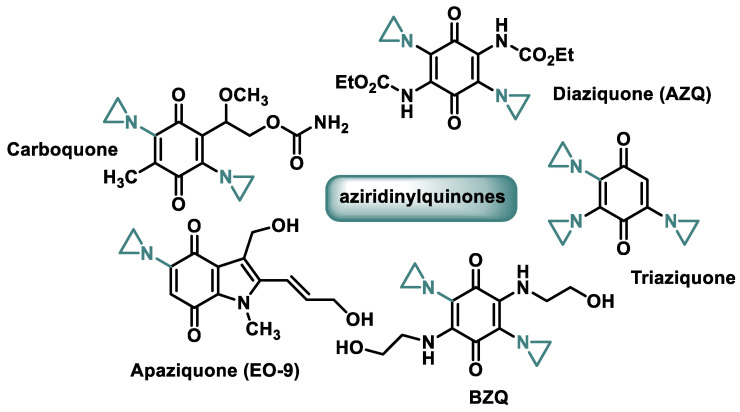
Chemical structure of Aziridinylquinones.

Antitumor compounds with two or three aziridine rings linked to a benzoquinone system can act as DNA bisalkylators and cross-linking agents and cross the blood–brain barrier due to their high ionization and reductively activated lipophilicity (Figure 31).

Diaziquone (AZQ) Diaziquone (AZQ) [82] was granted orphan drug status by the FDA in the early 1980s but showed no improvement over existing drugs. AZQ is a synthetic, lipophilic aziridine that can cross cell membranes and the blood–brain barrier. This drug has been used in CNS cancer and has shown therapeutic effect in these tumors. AZQ has also been used in the treatment of other solid tumors and leukemia.

Diazichon (AZQ) and carboquinone (carbazilquinone) have been tested in the treatment of various cancers but show low efficacy and toxicity. Triazichon (Oncovedex) was used clinically for the treatment of cancer in 1960. It is highly toxic to the walls of blood vessels and bone marrow, so it was replaced by other, more important and effective agents [83].

Apaziquone (EO9), a bioreductive drug with only one azidirine moiety in its structure, showed no activity in phase II clinical trials when administered intravenously, but in subsequent studies, it proved to be active and well tolerated in patients with superficial transitional cell carcinoma of the bladder after intravesical administration [84]. Approval was denied in 2016 after poor results in phase III clinical trials.

Aziridinylquinone BZQ was also the subject of several clinical trials in humans [72]. Mitomycin C and diaziquone (AZQ) are currently used in chemotherapy.

#### 6.1.3. Aziridines β-D-Galactopyranosides

**Figure 32:**
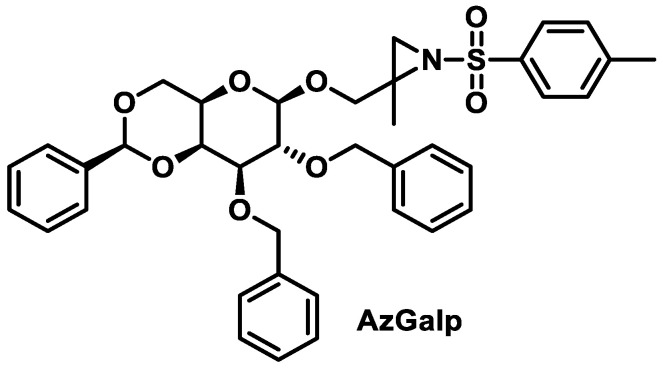
Chemical structure of AzGalp.

Calderón-Montaño et al., 2021, investigated various β-D-galactopyranoside-aziridine derivatives as anticancer agents. The best performing was 2-methyl-2,3-[N-(4-methylbenzenesulphonyl)imino] propyl 2,3-di-O-benzyl-4,6-O-(S)-benzylidene-β-D-galactopyranoside (AzGalp) (Figure 32), which has been shown to induce DNA damage [64].

### 6.2. Mechanism of DNA Alkylation by Aziridinylbenzoquinones

**Figure 33:**
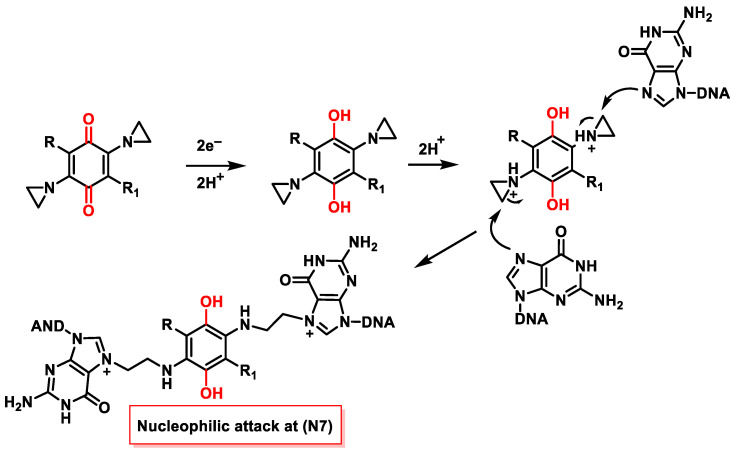
Reduction mechanism and structure of cross-links in DNA by aziridinylbenzoquinone derivatives.

The mechanism of DNA alkylation by aziridinylbenzoquinone derivatives involves bioreductive processes [64,85,86,87] due to the action of one- or two-electron reductases. Aziridinylbenzoquinones enter the cell in an inactive, oxidized form and are reduced to hydroquinone by enzymes in the cell, increasing the basicity of the aziridine groups and enhancing protonation until they become cytotoxic (Figure 33). This DNA damage increases when electron-donating groups are present on the benzoquinone ring, while the presence of electron-withdrawing functions or bulky groups at the C-6 position results in lower potency or inactive compounds [88].

One of these enzymes is DT-diaphorase (DTD, NQO1), a two-electron reductase that is located in the cell nucleus and whose level is elevated in many tumors [89]. The reduction is mainly due to the hypoxic conditions that often prevail in tumor cells [90]. Research is mainly aimed at finding new drugs that target this enzyme and have lower toxicity in normal tissue.

### 6.3. Inactivation of Aziridinylbenzoquinones

**Figure 34:**
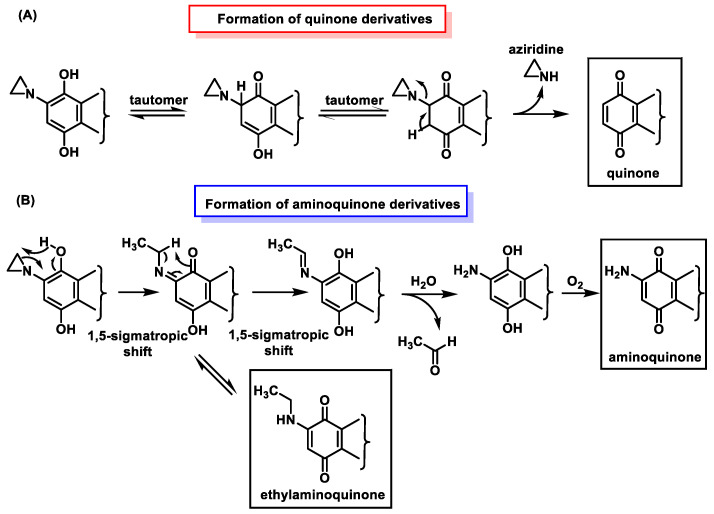
Inactivation of aziridinium benzoquinones (**A**) with loss of the aziridine ring and (**B**) initiated by a 1,5-sigmatropic shift.

In addition to alkylation, other reactions can also occur with aziridinylquinone derivatives. Other possible transformations that lead to the inactivation of these derivatives are shown in Figure 34—(A) Formation of quinone derivatives by loss of aziridine; (B) 1,5-sigmatropic hydrogen shift [91], which is then converted to ethylaminoquinone by tautomerism or to aminoquinone by a second 1,5-sigmatropic shift, followed by hydrolysis of the imine derivative in which acetaldehyde is lost; (C) formation of a quinone derivative by loss of aziridine; (D) formation of a quinone derivative by loss of aziridine, which then undergoes a second 1,5-sigmatropic hydrogen shift, followed by hydrolysis of the imine derivative, which undergoes loss of acetaldehyde.

**Figure 35:**
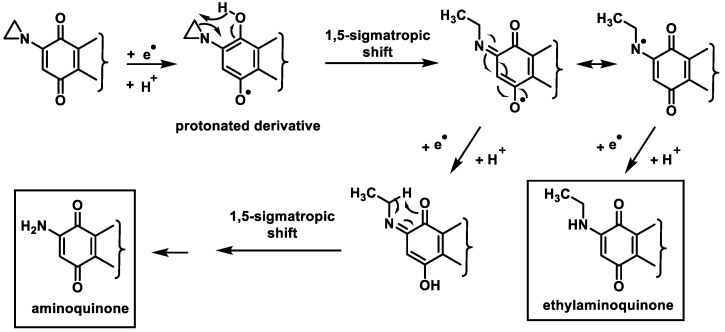
Inactivation of hydroquinone forms of aziridinylbenzoquinones by one-electron reduction.

The metabolic one-electron reduction of aziridinylquinones yields semiquinones. Their protonated derivatives undergo a 1,5-sigmatropic shift leading to inactive ethylaminoquinones and aminoquinones (Figure 35), as in the two-electron reduction [92].

## 7. Epoxides

The high reactivity of the epoxide ring towards nucleophilic groups in biomolecules is the basis for the use of ethylene oxide in side chains of compounds intended for the alkylation of DNA.

**Figure 36:**
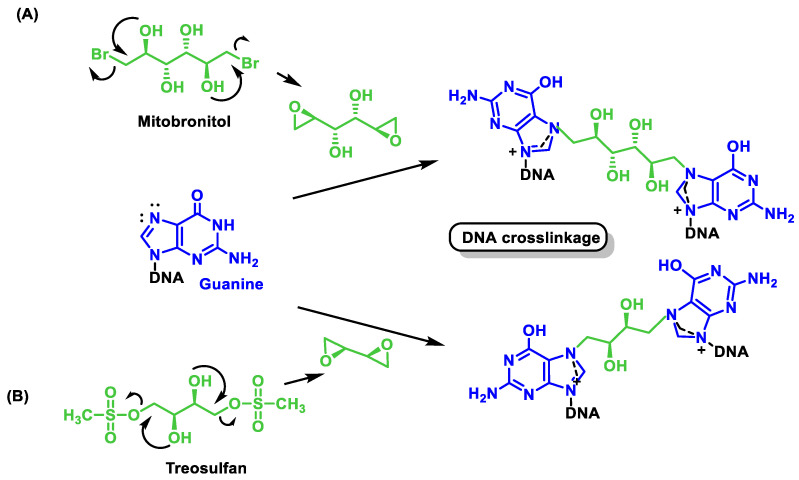
(**A**) DNA alkylation by mitobronitol and (**B**) DNA alkylation by treosulphan.

Treosulphan and mitobronitol are two prodrugs that undergo intramolecular double nucleophilic displacement to yield the diepoxide derivative. This compound is able to crosslink DNA by alkylating the guanine bases of DNA [93] (Figure 36).

Treosulphan is mainly used for the treatment of ovarian cancer. In addition, it has shown preclinical and clinical activity in some other solid tumors and hematological malignancies and is used for bone marrow ablation prior to stem cell transplantation and for the treatment of malignant melanoma and breast cancer. Mitobronitol is the biomolecular analog of mannitol and is used for myelosuppression prior to allogeneic bone marrow transplantation in accelerated chronic granulocytic leukemia [94].

## 8. Nitrosoureas

**Figure 37:**
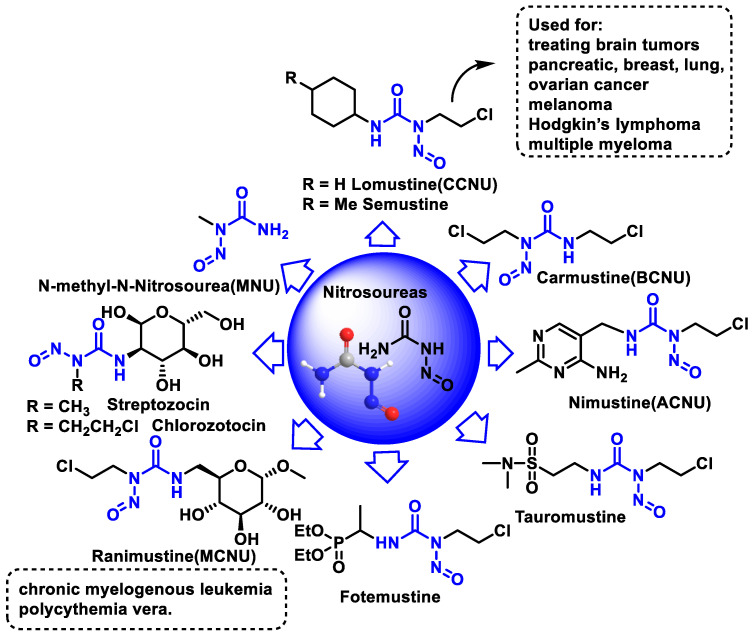
Chemical structure of the main nitrosoureas.

Antineoplastic agents derived from nitrosoureas are alkylating agents. The most important property is their ability to reach the brain, and they are used specifically to treat brain tumors [95]. All other alkylating agents lack this property and cannot reach the brain. Nitrosoureas can penetrate the brain because they are able to cross the so-called blood–brain barrier, a special area that prevents most drugs from reaching the brain. Nitrosourea derivatives such as carmustine and lomustine are effective in brain tumors, Hodgkin’s disease, and other lymphomas. Side effects of nitrosourea derivatives include nausea, vomiting, phlebitis, and suppression of hematopoiesis. Some of the nitrosourea-derived drugs that can treat certain brain tumors are shown in Figure 37.

1-Methyl-1-nitrosourea, the most important compound of the nitrosourea group, was found to be effective against intracerebrally implanted murine leukemia. However, it was found that the introduction of a 2-chloroethyl chain at the nitrogen-containing nitroso group led to a significant increase in the activity of the nitrosoureas. These 2-chloroethyl derivatives are lipophilic and can cross the blood–brain barrier and enable the treatment of brain tumors. In view of this activity, the nitrosoureas lomustine (CCNU), semustine, carmustine (BCNU), nimustine (ACNU), tauromustine, and fotemustine were synthesized, all of which are water-soluble but have significant toxicity problems.

Carmustine was tested in clinical trials in 1964 and approved by the FDA in 1977 for the treatment of various types of brain tumors (including glioma, glioblastoma multiforme, medulloblastoma, and astrocytoma), multiple myeloma, and lymphomas (Hodgkin’s and non-Hodgkin’s lymphoma).

A new formulation of carmustine with reduced systemic toxicity has been developed for the local treatment of brain tumors. The formulation, in the form of a slow-release “wafer” (Gliadel Wafer^®^; Polifeprosan 20 with carmustine), is implanted into the resection cavity remaining after surgical removal of the tumor. It was approved by the FDA in 1997 as an adjunct to surgery to prolong the survival of patients with recurrent GBM for whom surgical resection is indicated.

BCNU is a prodrug that breaks down to produce chloroethylalkylation chloroethyl residues that can form cross-links between DNA strands [96]. Carbamoylation of lysine residues in nucleoproteins via isocyanate intermediates may also play a role in BCNU’s anticancer mode of action [97].

Nimustine is used in combination with teniposide as second- or third-line chemotherapy for recurrent glioblastoma.

Streptozotocin (a glucosamine nitrosourea), a natural hydrophilic nitrosourea, was isolated from Streptomyces achromogenes (1967). This compound proved to be more effective and less toxic, was approved by the FDA in 1982 for the treatment of pancreatic islet cell cancer and marketed as Zanosar^®^, although its use is generally limited to patients whose cancer cannot be removed by surgery, and several analogs have been produced, such as chlorozotocin and ranimustine.

Streptozotocin differs from other nitrosoureas in that it does not cross the blood–brain barrier due to its high hydrophilicity and also has relatively low myelosuppression, as it penetrates less into the cells of the bone marrow.

Chlorozotocin has been shown in a phase II study to be as active against metastatic melanoma as other clinically used chloroethylnitrosoureas without causing bone marrow toxicity [98]. Chlorozotocin is probably carcinogenic to humans.

### Mechanism of Action of Nitrosoureas

**Figure 38:**
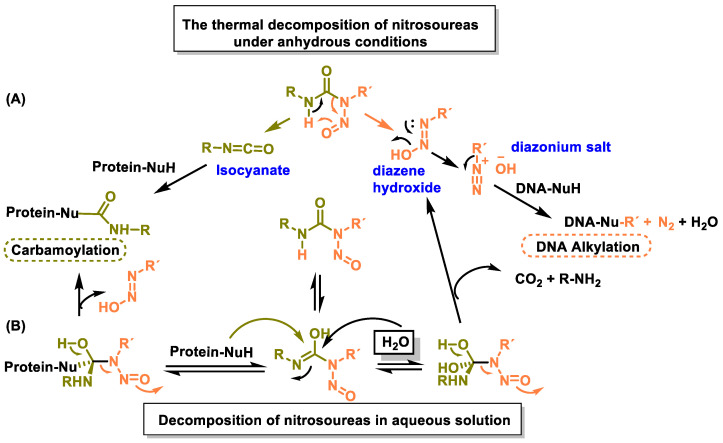
(**A**) Decomposition of nitrosoureas under anhydrous conditions. (**B**) In aqueous solution with the products of the alkylation of DNA and proteins.

The mechanism of action of nitrosoureas has been extensively studied. Nitrosoureas decompose spontaneously to form two electrophiles: an isocyanate and a diazene hydroxide. The diazene hydroxide produces a diazonium salt that alkylates the DNA, and this appears to be the main reaction responsible for the antitumor activity [99]. The isocyanate reacts with the amino groups of proteins to cause carbamoylation of proteins, which leads to inhibition of various DNA repair mechanisms. This fragmentation pathway was proposed based on studies of thermal decomposition of nitrosoureas under anhydrous conditions, but in aqueous solution the reaction proved to be more complex [71] (Figure 38).

**Figure 39:**
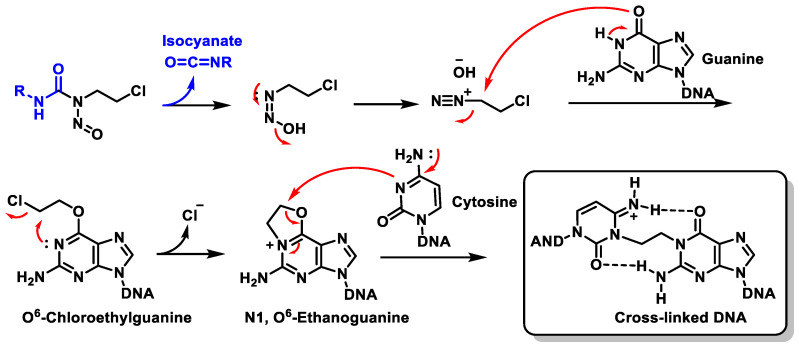
DNA cross-linking by nitrosoureas.

Almost all nitrosoureas contain a chloroethyl chain in the nitroside, which enables them to act as DNA cross-linkers. This monoalkylated product forms a 5-linked cyclic intermediate and then reacts with the N-3 atom of the cytosine unit on the complementary DNA strand to form the cross-linked end product (Figure 39).

An alternative mechanism would be the direct alkylation of DNA by the nitrosourea itself and not by the diazonium salt. Most drugs from the nitrosourea group are no longer in use and have been replaced by other alkylating agents.

## 9. Methanesulphonates

**Figure 40:**
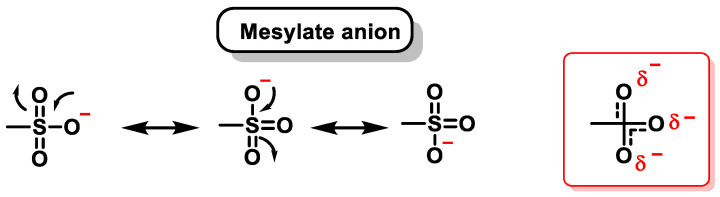
Mesylate ion resonant forms and average structure.

The mesylate is a good leaving group due to the delocalization of the negative charge between its three oxygen atoms (mesylate anion) (Figure 40).

**Figure 41:**
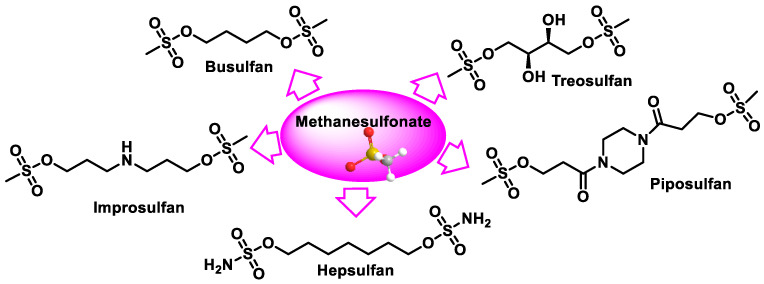
Methanesulphonate used as antitumor agents.

Several compounds containing two methanesulphonate groups separated by a hydrocarbon–carbon chain was tested as antitumor agents, and the compound with four carbons (busulphan) was found to have the optimal activity. The other methanesulphonate derivatives tested include piposulphan, improsulphan, hepsulphan, and treosulphan, a di-epoxide prodrug, which are shown in Figure 41.

Busulphan, 4-methylsulphonyloxybutylmethanesulphonate, is an antineoplastic agent from the class of bifunctional alkylating agents that has been used to treat various types of cancer since 1959. Busulphan is used to treat chronic myeloid leukemia and certain blood disorders such as polycythemia vera and myeloid metaplasia and is also used in some conditioning programs prior to bone marrow transplants [100,101,102,103]. In contrast to nitrogen mustard and nitrosoureas, busulphan mainly affects myeloid cells. In high doses, busulphan is used in combination with cyclophosphamide as part of a preparatory regimen for bone marrow transplants to treat childhood leukemias, lymphomas, and some solid tumors. Busulphan is toxic to lung tissue and this toxicity may limit the dose that can be used.

Close monitoring of blood counts is required in patients treated with busulphan. Hematologic toxicity, including severe neutropenia, leukopenia, thrombocytopenia, and anemia, is the dose-limiting factor for busulphan.

**Figure 42:**
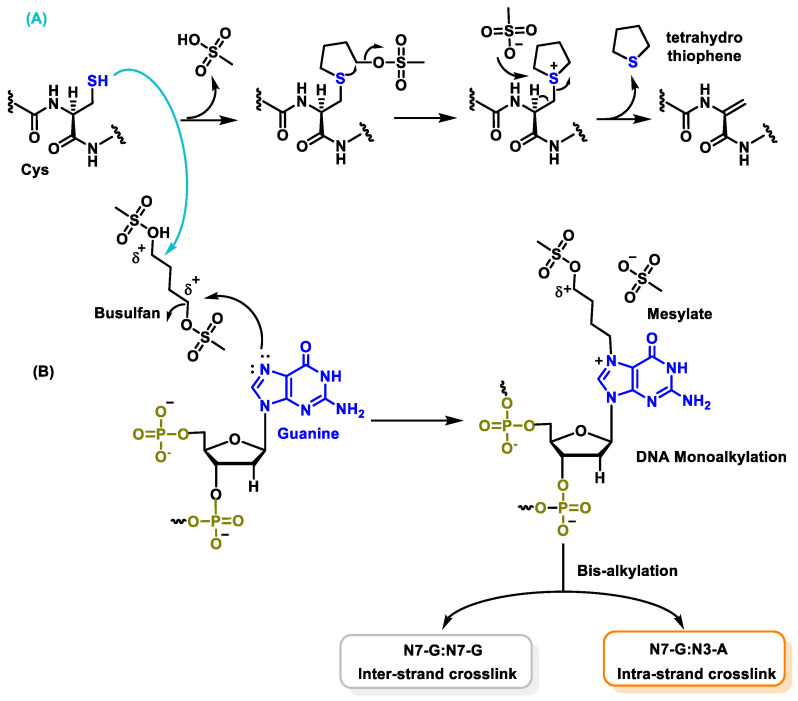
(**A**) Alkylation of cysteine residues by busulphan and (**B**) mechanism of DNA alkylation by busulphan.

In aqueous solutions, busulphan hydrolyzes to release methanesulphate groups and generates carbonium cations, which are reactive and can alkylate DNA and other proteins, leading to cytotoxicity. Busulphan reacts more readily with the thiol groups of amino acids, with cysteine, which involves a double nucleophilic attack of busulphan on the amino acid (Figure 42), and also with proteins as nitrogen mustard. In the final step of this reaction, elimination of the tetrahydrothiophene takes place and a C-C double bond is formed. The alkylation reaction depends on the concentrations of both busulphan and the target compound. Busulphan binds to DNA at the N-7 position of guanine. The alkylation of guanine with busulphan occurs via an SN2 mechanism [104]. The mechanism involves the attack of the nucleophile on the carbon containing the leaving group (mesylate). This carbon has a clearly positive polarity due to the electro-negativity of the sulphonate residue. Simultaneously with the attack on the nucleophile (N-7), the carbon-mesylate bond is cleaved, forming the monoalkylation product. The formation of cross-links between DNA strands has been demonstrated for busulphan cross-links within the DNA strand, mainly in the guanine–adenine sequence [102,105] (Figure 42).

Hepsulpham induced higher levels of DNA strand cross-linking than busulpham in three isolated samples from patients with chronic myeloid leukemia in blast crisis. However, busulphan caused a low number of DNA strand breaks in human cells.

Treosulphan in combination with fludarabine is a drug that is given to people before they undergo a bone marrow transplant from a donor, called an allogeneic hematopoietic stem cell transplant. It is used as a ‘conditioning treatment’ to cleanse the patient’s bone marrow and make room for the transplanted bone marrow cells, which can then produce healthy blood cells. It is used in both adult and pediatric patients older than one month with malignant neoplasms and benign diseases.

## 10. Triazenes

**Figure 43:**
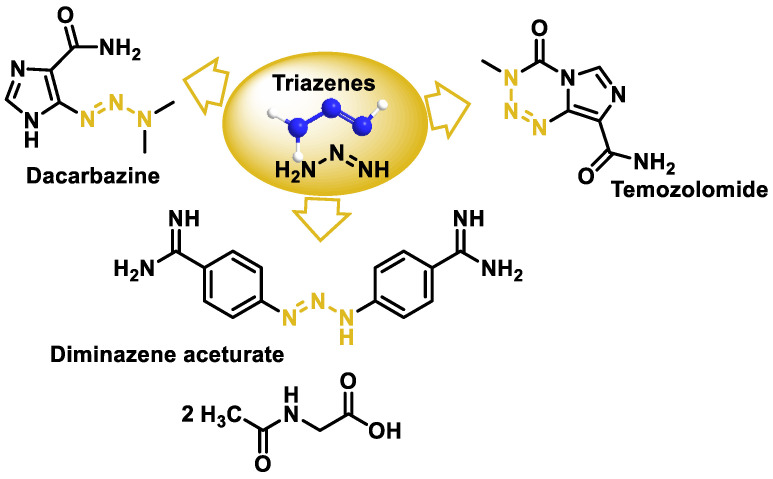
Current therapeutic use triazenes.

Triazenes are molecules that contain three contiguous nitrogen atoms in a linear arrangement. They have anticancer potential in many types of tumors such as leukemia, melanoma, and brain tumors as well as other therapeutic applications. The three triazenes used therapeutically are the anticancer drugs dacarbazine and temozolomide, and dia-minazole acetate, which is used in the treatment of trypanosomiasis in animals (Figure 43).

Dacarbazine (DAC) (5-(3,3-dimethyl-1-triazenyl) imidazole-4-carboxamide) belongs to the triazene group of alkylating agents used in cancer therapy [106,107,108,109]. DAC has a triazene group linked to the imidazole ring, which, in turn, is linked to the carboxamide group. It is an electrophilic agent that acts specifically in the S phase of the cell cycle. Dacarbazine was approved in the 1970s in the USA and France for the treatment of metastatic malignant melanoma, Hodgkin’s lymphoma, sarcoma [110], and pancreatic islet cell carcinoma and is used in combination with other cancer drugs in more extreme cases. It has been classified in the group of antitumor agents for which there is no safe exposure range [111].

**Figure 44:**
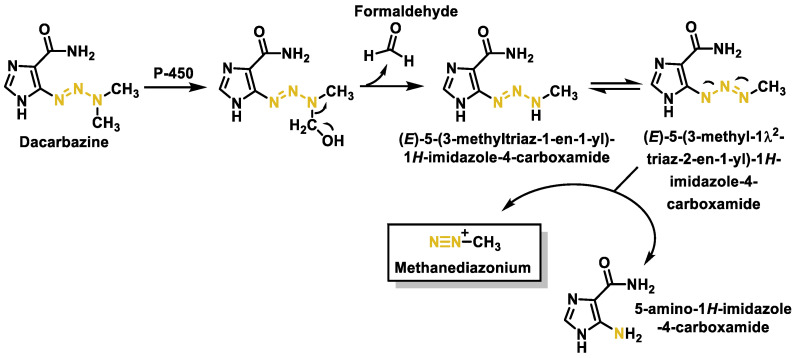
Generation of methanediazonium from dacarbazine.

It is a prodrug that is activated in the liver and its cytotoxic activity is due to the formation of methyldiazonium during its metabolism, which methylates DNA [112]. Methyldiazonium has a very short half-life of about 0.4 s in aqueous solution. A mechanism for this process is summarized in Figure 44, where activation of dacarbazine by metabolic oxidative demethylation with formation of formaldehyde and (E)-5-(3-methyltriaz-1-en-1-yl)-1H-imidazole-4-carboxamide from the tautomer generates the reagent methyldiazonium. The formation of this cation is considered to be the main mechanism of its antitumor effect (Figure 44).

**Figure 45:**
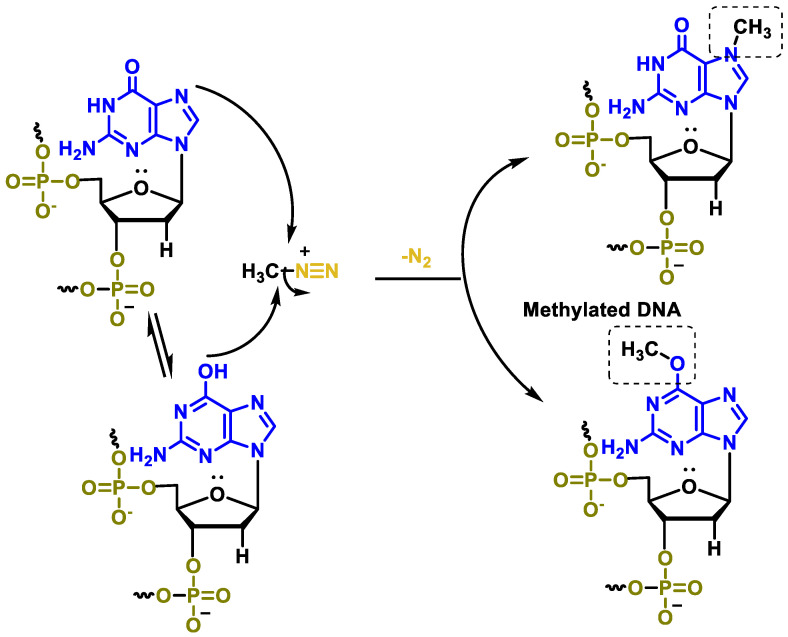
Mechanism of DNA methylation by Dacarbazine.

The most important methylation reaction takes place at the N-7 guanine atom and is relatively non-toxic. Methylation of the O-6 guanine also occurs and is considered to be the primary cytotoxic mechanism [113] (Figure 45).

**Figure 46:**
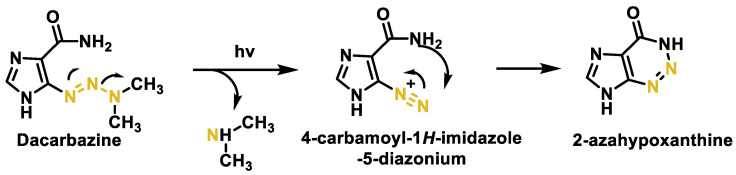
Dacarbazine photodegradation products.

Disadvantages of dacarbazine are: (i) toxicity, (ii) excessive hydrophilicity leading to slow and incomplete oral absorption, necessitating intravenous administration, and (iii) its high photosensitivity with a very short half-life (about 30 min). This antineoplastic drug is excreted in the urine and its half-life in the body is 5 h. Dacarbazine is unstable in aqueous solution and is spontaneously degraded in the light, decomposing to 5-diazomidazole-4-carboxamide and di-methylamine and also undergoing a structural rearrangement to 2-azahipoxanthine [114,115] (Figure 46).

5-Diazomidazole-4-carboxamide can attack nucleophilic groups of DNA. However, the degradation products of dacarbazine probably do not contribute significantly to its cytotoxicity, although they may be involved in the painful local burning sensation on intravenous injection and in systemic problems associated with the drug.

Temozolamide (TMZ) was synthesized in 1980 as part of a series of new imidazo-tetrazinones to address the problems caused by the use of dacarbazine and has replaced dacarbazine in the clinic. It is a cytotoxic alkylating prodrug used for the treatment of glioblastoma multiforme [116,117] {Stevens, 1984 #12198}. Temozolomide, a methylating agent, was approved by the FDA in the USA in 1999 for the treatment of refractory anaplastic astrocytoma. The European Agency for the Evaluation of Medicinal Products approved the drug in 1998 for (i) progressive or recurrent glioblastoma multiforme after standard surgery and (ii) in 1999 for anaplastic astrocytoma, which is also resistant to treatment. Importantly, temozolomide is one of the few drugs approved specifically for a brain tumor and the first that can be administered orally.

**Figure 47:**
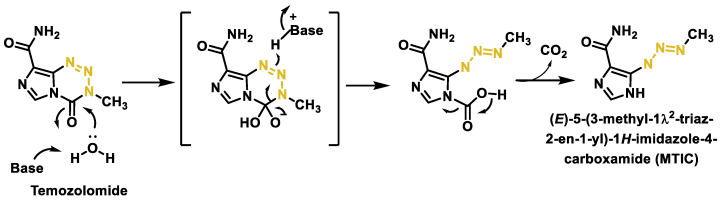
Temozolomide hydrolysis.

The chemotherapeutic activity is achieved by converting temozolamide to the same intermediate (5-methyltriazenoimidazole-4-carboxamide) as dacarbazine, but in the case of temozolomide, the bioactivation process involves a non-enzymatic hydrolysis reaction followed by decarboxylation (Figure 47).

The methyldiazonium ion formed during the degradation of MTIC mainly methylates the guanine residues in the DNA molecule, leading to the formation of O6- and N7-methylguanine. This aberrant DNA can no longer be corrected by the cellular machinery and ultimately leads to cell death through apoptosis. In addition, unlike dacarbazine, this drug can be administered both parenterally and orally. The main problem with this drug is bone marrow toxicity.

## 11. Fate and Effects of Anticancer Drugs in the Environment

Cytotoxic drugs, i.e., cancer drugs used in chemotherapy, are administered in European countries in the order of tons per year. After administration, these compounds are excreted by the chemotherapy patient either in their original form or as a product of the metabolization of the original compound into wastewater and subsequently discharged into rivers, seas, and oceans and can enter the environment via wastewater treatment plants, where they can have harmful effects. Due to their physicochemical properties, they can also be adsorbed in sewage sludge. The incidence of cancer has increased in recent years and is expected to continue to rise, as is the consumption of cytotoxic drugs. Therefore, their control in the environment is of utmost importance [118].

Assessing the risk posed by cytotoxic drugs is of particular interest as they are cytotoxic and, in many cases, carcinogenic. There is currently little data on the occurrence and effects of cytotoxic drugs in the environment.

The release of cytotoxic drugs used in chemotherapy has received less attention than other pharmaceuticals, although they may have cytotoxic, genotoxic, mutagenic, carcinogenic, endocrine, and/or teratogenic effects on various organisms.

The main sources of these effluents are hospital effluents, effluents from drugs excreted during outpatient treatment, and drug manufacturers. The immediate consequence is the release into the aquatic and terrestrial environment, even in minute quantities. The removal efficiency of wastewater treatment varies and depends on both the physicochemical properties of these by-products and the treatment process used [119].

In the 1990s, major efforts were made to investigate the occurrence, phase, and potential risks of human pharmaceuticals in the environment. Several large-scale collaborative programs have been conducted in Europe and the US, such as ‘Poseidon’ (EU, 2001–2004), Norman (EU, 2005–2008), PILLS (EU, 2007–2012), and Emerging Contaminants in the Environment (US Geological Survey, 2007–2011). The most recent PHARMAS (EU, 2011–2013) and CytoThreat (EU, 2011–2013) specifically target antibiotics and cancer drugs.

There is a need to sensitize both the scientific community and the general public to the potential of anticancer drugs as pollutants of increasing concern in aquatic and terrestrial ecosystems [120].

Antineoplastic drugs (ANPs) have raised concerns about their safe remediation. An excellent review paper by Abhilash Kumar Tripathi and co-authors provides an overview of the environmental sources of ANP agents, focusing on the remediation methods currently in use [121].

Other necessary considerations include the solid cytotoxic wastes generated during the preparation, reconstitution, and administration of these drugs to chemotherapy patients. There are no suitable treatment technologies for this waste in most parts of Spain, so it has to be transported to France and/or Germany for incineration, resulting in the emission of toxic and greenhouse gasses.

## 12. Antimicrobial Peptides

The search for more effective and less toxic cancer treatments has driven research into numerous innovative therapeutic strategies. Among these, antimicrobial peptides (AMPs) have emerged as a promising class of agents with potent anticancer properties [122]. Originally identified for their role in innate immunity and their ability to fight microbial infections, AMPs have shown a broad spectrum of biological activities that also have anticancer effects. These peptides exert their anticancer effects primarily through the selective destruction of cancer cell membranes, induction of apoptosis, and modulation of immune responses, making them highly versatile and potent candidates for cancer therapy [123]. Their unique mechanism of attacking cancer cells while sparing normal cells offers a significant advantage over conventional chemotherapeutic agents, which often result in significant collateral damage to healthy tissue [124,125].

Antimicrobial peptides (AMPs) are a critical component of the innate immune system and serve as one of the first lines of defense against a broad spectrum of pathogens, including bacteria, viruses, fungi, and parasites [126]. These small, naturally occurring peptides are ubiquitous in a variety of species, from microorganisms to humans, underscoring their evolutionary importance [126]. The discovery of AMPs can be traced back to the early 20th century, when scientists first discovered the antimicrobial properties of lysozyme, an enzyme found in egg white and human tears [127]. But it was not until the late 1980s that the field of AMPs gained prominence after magainin were isolated and characterized from the skin of the African clawed frog *Xenopus laevis* [128]. This groundbreaking work highlighted the strong antimicrobial activity of these peptides and initiated extensive research into their potential therapeutic applications.

AMPs are characterized by their amphipathic nature, which enables them to interact with and destroy microbial membranes [129]. Structurally, they typically consist of 12–50 amino acids and can adopt various conformations, including alpha helices, beta sheets, and extended or looped structures [129,130]. The ability of AMPs to attack and destroy the lipid bilayers of microbial membranes, while mammalian cells are generally spared, is attributed to differences in membrane composition [122]. This selectivity is crucial for their role in host defense and represents a significant advantage over conventional antibiotics, which often cause problems with resistance and collateral damage to host tissue [129].

Beyond their antimicrobial properties, AMPs have attracted attention for their diverse role in modulating the immune response, promoting wound healing and even their anticancer activity. In the context of cancer therapy, AMPs have been shown to selectively target cancer cells through mechanisms similar to those used to fight microbes [122]. Cancer cells often have particular membrane characteristics, such as a higher proportion of negatively charged phospholipids, which make them susceptible to AMP-induced membrane disruption. In addition, AMPs can induce apoptosis in cancer cells and modulate the tumor microenvironment to enhance antitumor immunity. These properties make AMPs promising candidates for novel cancer therapies, either as stand-alone treatments or in combination with other therapeutics [122].

The potential of AMPs in cancer therapy has generated considerable interest in the scientific community, particularly with regard to their use in combination with other therapeutic modalities [131]. One of these promising combinations is with DNA alkylating agents [132,133,134,135]. DNA alkylation involves the attachment of alkyl groups to DNA molecules, resulting in DNA damage and inhibition of cancer cell proliferation. Alkylating agents, such as cyclophosphamide and cisplatin, have been cornerstones of cancer treatment for decades [60]. However, their use is often limited by the development of resistance and severe side effects [136]. The combination of DNA alkylating agents with AMPs offers a novel approach to overcome these limitations by increasing the efficacy and specificity of the attack on cancer cells [137,138]. The reason for this combination lies in the complementary mechanisms of action of AMPs and alkylating agents. While alkylating agents have a direct genotoxic effect on cancer cells, AMPs can enhance this effect by compromising the integrity of cancer cell membranes, enabling better intracellular uptake of the drug and promoting apoptosis. In addition, AMPs can modulate the tumor microenvironment by enhancing the immune response against cancer cells, potentially attenuating the immunosuppressive effects often associated with alkylating chemotherapy. This synergistic interaction promises not only better therapeutic outcomes but also a reduction in the required doses of alkylating agents and thus a minimization of their undesirable side effects [2,139,140,141].

## 13. Mechanisms of Action of AMPs in Cancer Cells

Antimicrobial peptides (AMPs) exhibit a versatile mode of action against cancer cells by utilizing their innate properties to attack and destroy malignant cells. One of the most important mechanisms by which AMPs exert their anticancer effect is by destroying the membranes of cancer cells [122]. Cancer cells often have special membrane characteristics, including a higher content of negatively charged phospholipids and an altered lipid composition compared to normal cells. This makes them particularly susceptible to the amphipathic and cationic nature of AMPs. Upon interaction with the cancer cell membrane, AMPs can penetrate the lipid bilayer and form pores or degrade the membrane. This disruption impairs the integrity of the cell membrane and leads to cell lysis and cell death [142].

In addition to the direct disruption of the membrane, AMPs can also trigger apoptosis in cancer cells. Apoptosis, or programmed cell death, is a tightly regulated process that eliminates damaged or unwanted cells without causing inflammation. AMPs can trigger apoptosis via different pathways, including the mitochondrial pathway and the death receptor pathway. In the mitochondrial pathway, AMPs can cause the release of cytochrome c from the mitochondria into the cytosol, which subsequently activates caspases, the enzymes responsible for carrying out apoptosis. Alternatively, AMPs can interact with cell surface receptors to activate the death receptor pathway, leading to the formation of the death-inducing signaling complex (DISK) and activation of caspase-8. These pathways culminate in the cleavage of important cellular proteins and the eventual destruction of the cell.

AMPs can not only trigger apoptosis but also modulate the immune response, creating a hostile environment for cancer cells [143]. AMPs are known to have immunomodulatory properties. These include the ability to attract immune cells to the tumor site, promote the maturation and activation of dendritic cells, and enhance the presentation of tumor antigens. This immunomodulation can stimulate both the innate and adaptive immune response, leading to a stronger attack on the cancer cells [144]. By altering the tumor microenvironment, AMPs can help overcome the immunosuppressive barriers that often protect tumors from surveillance and destruction by the immune system [122,145].

In addition, some AMPs have been found to inhibit angiogenesis, the process by which tumors establish their own blood supply to sustain growth and metastasis [122,146]. Angiogenesis is a crucial step in tumor progression, and inhibiting it can supply the tumor with essential nutrients and oxygen [147]. AMPs can interfere with angiogenic signaling pathways by reducing the expression of pro-angiogenic factors such as vascular endothelial growth factor (VEGF) and disrupting the formation of new blood vessels [148,149]. This anti-angiogenic effect not only inhibits tumor growth but also limits the potential for metastasis [150].

## 14. Advantages of AMPs and Their Synergy with Alkylating Agents

**Figure 48:**
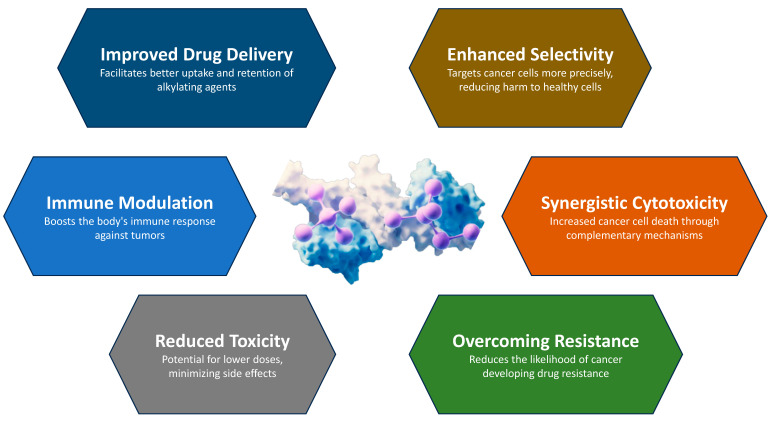
Advantages of antimicrobial peptides in combination with alkylating agents for cancer therapy.

One of the main problems with alkylating agents is their lack of selectivity, which results in damage to both cancer cells and healthy cells. This non-specificity leads to a number of serious side effects, including myelosuppression (bone marrow suppression), gastrointestinal toxicity, and an increased risk of secondary malignancies [151]. These side effects may limit the dose of alkylating agents that can be safely administered to patients, reducing their overall efficacy [136]. The membranes of cancer cells often contain higher levels of negatively charged phospholipids and other unique lipid components that attract the cationic and amphipathic nature of AMPs [122,152]. This selectivity minimizes collateral damage to healthy cells and reduces the side effects commonly associated with conventional chemotherapy [153] (Figure 48).

AMPs also exhibit a broad spectrum of anticancer mechanisms, making it difficult for cancer cells to develop resistance. Their ability to disrupt cell membranes, induce apoptosis, modulate immune responses, and inhibit angiogenesis provides a versatile approach to destroying cancer cells. By attracting immune cells to the tumor site and promoting the activation and maturation of dendritic cells, AMPs can stimulate both the innate and adaptive immune systems. This immune modulation can create a more hostile environment for cancer cells, further enhancing the anticancer effects of AMPs and also helps to prevent the emergence of resistant cancer cell populations, which is a major problem with single-target chemotherapeutics [154].

The natural origin of many AMPs and their relatively simple structure also facilitate their synthesis and modification, enabling the development of a wide range of AMP derivatives with improved stability, potency, and selectivity. Advances in peptide engineering and delivery systems have further enhanced the therapeutic potential of AMPs, making them more suitable for clinical use [142]. These properties make AMPs promising candidates for the development of novel cancer therapies, either as stand-alone treatments or in combination with existing chemotherapeutics to increase efficacy and reduce toxicity.

The combination of AMPs with alkylating agents can improve the efficacy of cancer therapy by several mechanisms (Table 2): (i) by enhancing DNA damage, since alkylating agents act by attaching alkyl groups to DNA and AMPs can enhance this effect by interfering with DNA repair mechanisms, making cancer cells more susceptible to damage by alkylating agents; (ii) by modulating the tumor microenvironment, affecting angiogenesis (formation of new blood vessels) and immune responses, making tumor cells more susceptible to the cytotoxic effects of alkylating agents; (iii) overcoming drug resistance by disrupting cell signaling pathways that cancer cells use to escape the effects of DNA damage; and (iv) activating signaling pathways that lead to apoptosis (programmed cell death) and complement the cytotoxic effects of alkylating agents. This dual approach may lead to more effective elimination of cancer cells.

## 15. Technological Advances in the Administration of AMPs and Alkylating Drugs

**Figure 49:**
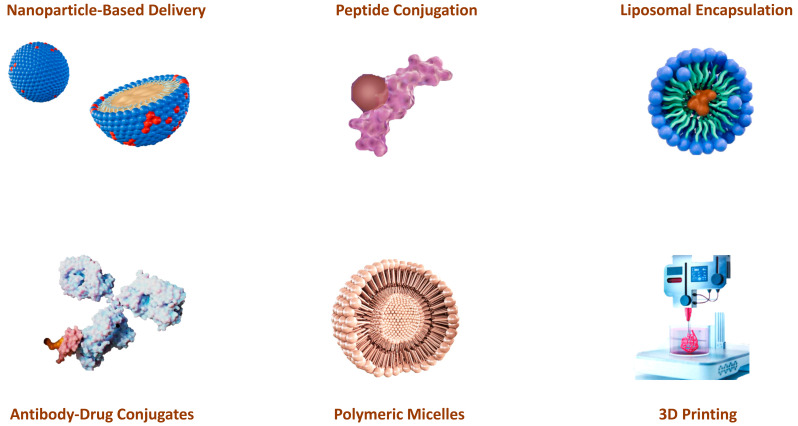
Technological advances in the administration of AMPs and alkylating drugs for cancer therapy.

The development of effective delivery systems is critical to the therapeutic success of antimicrobial peptides (AMPs) and alkylating agents in cancer treatment (Figure 49). One of the most important advances in AMP delivery is the use of nanoparticle-based systems [161]. Nanoparticles can be engineered to encapsulate AMPs, protect them from enzymatic degradation, and improve their stability in the bloodstream [162,163]. These nanoscale carriers can be functionalized with targeted ligands that recognize and bind to specific markers on the cancer cells to ensure that the AMPs are delivered precisely to the tumor. This targeted delivery not only increases the therapeutic efficacy of the AMP, but also minimizes off-target effects and reduces systemic toxicity. Various nanoparticle platforms, including liposomes, polymeric nanoparticles, and metallic nanoparticles, have been evaluated for their ability to effectively deliver AMPs to tumors [164].

Similarly, advances in the delivery of alkylating agents have focused on improving their selectivity and reducing their toxicity. Liposomal encapsulation is one of these promising strategies. Liposomes are spherical vesicles consisting of lipid bilayers that can encapsulate hydrophilic and hydrophobic drugs. Encapsulation of alkylating agents in liposomes may help to protect them from premature degradation and reduce their systemic toxicity by enhancing their accumulation in tumor tissue through the effect of enhanced permeability and retention (EPR). This effect takes advantage of the leaky vasculature and poor lymphatic drainage of tumors to preferentially deliver liposomal encapsulated drugs to the tumor [165,166,167].

Another significant advance is the development of antibody–drug conjugates (ADCs), which combine the targeting ability of monoclonal antibodies with the potent cytotoxic activity of alkylating agents. ADCs are designed to bind specifically to antigens expressed on the surface of cancer cells, delivering the alkylating agent directly to the tumor and sparing normal tissue. This targeted approach maximizes the therapeutic index of alkylating agents and increases their efficacy while minimizing adverse effects [168,169].

Polymeric micelles have also been used to enhance the delivery of AMP and alkylating agents. These self-assembling nanocarriers can encapsulate hydrophobic actives in their core and form a hydrophilic shell that improves the solubility and stability of the encapsulated actives. Polymeric micelles can be engineered to release their payload in response to specific stimuli, such as changes in pH or temperature, ensuring controlled and sustained drug release at the tumor site [164,170,171,172].

In addition to nanoparticle-based systems, peptide conjugation strategies have been developed to improve the delivery of AMPs. Conjugation of AMPs with cell-penetrating peptides (CPPs) can facilitate their translocation across cell membranes, improving their intracellular release and therapeutic efficacy. CPPs can be designed to recognize and bind to specific receptors on cancer cells, ensuring targeted delivery of AMPs and enhancing their anticancer effects [173,174]. For example, a combination of the cell-penetrating peptide octoarginine–oxaliplatin was developed to efficiently and rapidly transport oxaliplatin into colon cancer cells. The in vivo study in mice showed that the compound successfully suppressed tumor development and had a significantly strong anticancer effect [175]. Similarly, the complex of the cell-penetrating peptide Tansportan-10 and cisplatin produced a stronger effect on the cancer cell lines (HeLa, OS143B) compared to that observed after separate treatment [176]. Conjugation of cell-penetrating peptides with cobalt complexes also showed superior efficacy against the HepG2 liver cancer cell line compared to the original Co(III) complex [177]. The combination of cell-penetrating peptides with nanotechnology is a promising strategy. Encapsulation of the cell-penetrating peptide EIP103, which specifically targets the nucleus, in PEG-PE micelles showed significant inhibitory effects in H446 and A549 cells and demonstrated a promising therapy to improve the clinical treatment of lung cancer [178].

Advances in 3D printing technology have opened new avenues for the local delivery of AMPs and alkylating agents; 3D-printed scaffolds and hydrogels can be designed to release these agents in a controlled manner and ensure sustained therapeutic concentration at the tumor site. This local delivery approach can minimize systemic exposure and reduce the risk of adverse effects [179,180,181]. These advances demonstrate the importance of continuing drug delivery research and development to fully realize the therapeutic potential of AMPs and alkylating agents.

## 16. Can AI Tools Accelerate Current Research into Alkylating Agents?

The question of whether artificial intelligence (AI) can accelerate research into alkylating agents in cancer treatment is a hot topic that is increasingly being answered in the affirmative.

Alkylating agents are a class of chemotherapeutic agents that damage the DNA of cancer cells. However, their use is limited by significant side effects and the emergence of tumor resistance. This is where AI can make a decisive difference.

AI can accelerate research by discovering new drugs by analyzing large amounts of data. AI can analyze huge databases of molecules to identify new chemical structures with potential antitumor activity and through molecular modeling that simulates the interaction between molecules and proteins [182]. AI can predict the efficacy and toxicity of new drugs, reducing the time and cost of preclinical trials [183].

It can also help optimize treatments, such as personalizing therapies by analyzing each patient’s genomic data and identifying the most appropriate treatments that minimize side effects. It can also be used to predict response to treatment by analyzing medical imaging and clinical data so that therapy can be better tailored [184]. Another potential area of application is understanding resistance mechanisms and identifying potential biomarkers that predict the occurrence of resistance to alkylating agents [185].

In the negative perception, AI relies on large amounts of high-quality data. Therefore, more comprehensive and standardized databases need to be developed, and tools need to be developed to understand how the models arrive at their conclusions [186].

AI therefore has the potential to revolutionize alkylating agent research and ultimately improve outcomes for cancer patients. However, it is important to address the existing challenges and encourage collaboration between researchers from different disciplines to achieve the most out of this technology [187].

## 17. Conclusions

In the last thirty years, more and more drugs have been used to treat tumors, as genetic and environmental factors have led to an increased incidence of cancer in the human population. The structure and dynamics of DNA are strongly influenced by the alkylation of its bases. Alkylation prevents DNA replication and RNA transcription of the affected DNA molecule. It also leads to DNA fragmentation through hydrolytic reactions and through the action of repair enzymes that attempt to remove the alkylated bases. Alkylation also leads to mismatching of nucleotides by disrupting the normal hydrogen bonds between the bases.

Current research on alkylating agents should focus on the development of more selective alkylating agents with fewer side effects and adequate bioavailability. New derivatives of nitrogen mustard continue to be investigated for improved efficacy and reduced toxicity with combinations of nitrogen mustard with monoclonal antibodies and small molecule targeted agents.

The increasing use of these drugs and their degradation products has resulted in their appearance in water bodies around the world, impacting ecosystems, polluting the environment, and having negative effects on biota.

The combination of antimicrobial peptides (AMPs) and alkylating agents is a promising strategy towards more effective and targeted cancer therapies. AMPs offer significant advantages over conventional chemotherapeutics with their versatile mechanisms of action, including membrane disruption, apoptosis induction, immune modulation, and anti-angiogenic properties. These peptides selectively target cancer cells while sparing healthy tissue, minimizing systemic toxicity and reducing the risk of resistance development. Meanwhile, alkylating agents remain central to cancer treatment due to their ability to cause DNA damage and inhibit cell proliferation, albeit with significant limitations such as non-specific toxicity and the potential for resistance.

The combination of AMPs with alkylating agents capitalizes on the complementary strengths of these agents and may improve therapeutic efficacy through synergistic interactions. Technological advances in drug delivery, such as nanoparticle-based systems, antibody–drug conjugates, and 3D printing technologies, have further optimized the delivery of AMPs and alkylating agents to tumor sites, improving their bioavailability and therapeutic outcomes.

Combining AMPs with alkylating agents can also improve the efficacy of cancer therapy by enhancing DNA damage, modulating the tumor microenvironment, overcoming drug resistance, and activating signaling pathways that lead to apoptosis. These efforts may yield a more effective elimination of malignant cells.

While challenges such as resistance mechanisms, off-target effects, and optimal dosing strategies remain unresolved, ongoing research and clinical trials continue to explore the full potential of this dual mode of action. Future directions include the development of novel AMP derivatives, personalized medicine approaches, and combination therapies tailored to the individual patient profile. Ultimately, the convergence of AMPs and alkylating agents represents a promising paradigm shift in cancer therapy, offering hope for more effective treatments and better outcomes for patients with this difficult disease.

## Figures and Tables

**Table 1:** Classification of antineoplastic drugs according to the target and mechanisms of action.

Category	Mechanism of Action	Examples
Alkylating Agents	Act on the entire reproduction process; most effective during the DNA synthesis phase (S).	Derivatives of Nitrogen Mustards: cyclophosphamide, chlorambucil, ifosfamide. Tetrazenes–Triazenes: dacarbazine. Derivatives of Platinum: cisplatin, carboplatin, oxaliplatin.
Medications that Affect the Entire Process of Reproduction	Interfere with various stages of the cell cycle and reproduction process.	Nitrosureas: carmustine (bcnu), lomustine (ccnu), estramustine.
Antitumor Antibiotics	Interfere with DNA duplication and alter the membrane surrounding the cells.	Anthracyclines: doxorubicin, daunorubicin, epirrubicin, idarubicin, mitoxantrone. Others: bleomycin, mitomycin c, dactinomycin.
Antimetabolites	Act on the S phase (DNA synthesis), interfere with DNA and RNA.	Folic Acid Analogues: methotrexate. Pyrimidine Analogues: fluorouracil, cytarabine, gemcitabine, capecitabine. Purine Analogues: fludarabine, mercaptopurine, cladribine.
Campotecin Derivatives	Inhibit topoisomerase I, leading to DNA damage.	Irinotecan, topotecan.
Mitotic Inhibitors	Act during the M phase (mitosis), inhibit or stop mitosis, or inhibit enzymes needed for cell reproduction.	Vinca Alkaloids: vincristine, vinblastine, vindesine, vinorelbine. Taxoids: paclitaxel, docetaxel. Epipodophyllotoxins: etoposide, teniposide.
Other Mechanisms of Action	Affect tumors with hormone dependence or stimulate the immune system to attack cancer cells.	Hormones: diethylbestrol. Antiandrogens: flutamide, nilutamide, cyproterone. Antiestrogens: tamoxifen, anastrazole. Gonadotrophin Agonists: leuprolide, goserelin, buserelin, triptorelin. Progestagens: megestrol, medroxyprogesterone.
Immunotherapy	Stimulates the immune system’s natural defenses to destroy abnormal cells.	Interferons: interferon alpha 2a, alpha 2b. Monoclonal Antibodies: rituximab, cetuximab, trastuzumab.

**Table 2:** Synergistic mechanisms of AMPs and alkylating agents in cancer therapy.

Mechanism	Description	Reference
Membrane Disruption	AMPs disrupt cancer cell membranes, leading to cell lysis, facilitating the entry of alkylating agents.	[155]
DNA Damage	Alkylating agents cause DNA strand breaks, facilitated by AMPs.	[156]
Immune Modulation–Induction of Apoptosis	AMPs trigger apoptosis, which can be amplified by alkylating agents.	[157,158]
Anti-Angiogenesis	AMPs inhibit angiogenesis, which complements the cytotoxic effects of alkylating agents.	[159]
Overcoming Drug Resistance	AMPs reduce the potential for drug resistance to alkylating agents.	[160]
